# Epidemiology, Etiopathogenesis, Diagnosis, and Treatment of Male Infertility—Current Trends and Future Directions: A Narrative Review

**DOI:** 10.3390/medicina62030545

**Published:** 2026-03-14

**Authors:** Farooq Ahmed Wani

**Affiliations:** Department of Pathology, College of Medicine, Jouf University, Sakaka 72388, Saudi Arabia; fawani@ju.edu.sa; Tel.: +966-593750779

**Keywords:** male infertility, semen analysis, sperm DNA fragmentation, assisted reproductive technologies, idiopathic infertility

## Abstract

*Background and Objectives:* Male infertility has emerged as a growing global health concern, contributing to 20–30% of all infertility cases. It is a multifactorial condition, arising from genetic, endocrine, structural, environmental and lifestyle factors. This narrative review synthesizes current evidence on epidemiology, diagnostic advances and therapeutic strategies while highlighting emerging trends and research priorities. *Materials and Methods:* This review adheres to SANRA guidelines. Literature was sourced from PubMed, Saudi Digital Library, Google Scholar, and PsycINFO using MeSH terms including “Male Infertility,” “Diagnosis,” “Treatment,” and “Epidemiology.” *Results:* Diagnostic evaluation of male infertility includes clinical assessment, advanced semen analysis, imaging techniques, hormonal assays and molecular testing. Despite significant advances in the evaluation of male infertility, idiopathic causes (30–40%) remain challenging. Management strategies include lifestyle modifications, medical therapies including hormones and drugs, surgical interventions, and assisted reproductive technologies (ARTs). However, outcomes remain suboptimal in idiopathic and severe cases, particularly regarding sperm DNA fragmentation and environmental exposures. *Conclusions:* Substantial knowledge gaps exist in male infertility, particularly in idiopathic cases, molecular mechanisms of environmental pollutants, and long-term ART offspring outcomes. Future research priorities include: (1) molecular and epigenetic biomarkers for improved diagnosis and prognosis; (2) environmental exposure assessment and mitigation strategies; (3) metabolomics-guided personalized therapies; (4) regenerative medicine approaches including spermatogonial stem cell therapy; and (5) multidisciplinary integrative care models. Addressing these gaps through coordinated research and clinical innovation is essential for improving male reproductive health globally.

## 1. Introduction

Infertility is defined by the World Health Organization (WHO) and other societies as the inability to achieve pregnancy after 12 months or more of unprotected and regular sexual intercourse [1,2,3]. Infertility is categorized into primary and secondary infertility. As per the WHO report, infertility has become a global health concern, affecting millions of males and females of reproductive age; an estimated 15% of couples have been affected by this global health problem [2].

The psychosocial consequences imposed by the inability to procreate encompass multiple psychological sequelae, including altered sexual desire, other sexual dysfunction, clinical depression, anxiety disorders, loss of self-esteem, strained relationships, increased likelihood of divorce, social stigmatization, and in severe cases, suicidal tendencies [4,5,6]. Well-documented associations have been reported between infertility and eating disorders, body image disturbances, and marital breakdown, especially in pronatalist cultural contexts [5,7]. The stigma associated with infertility especially in numerous patriarchal societies across the globe disproportionately attributes reproductive failure to female partners, thereby delaying proper andrological evaluation and prolonging delays in diagnosis and treatment [8,9].

Infertility has been attributed to a wide range of etiological factors that reflect a complex interplay of female, male, and combined factors underscoring the complexity of its underlying pathophysiology. Well-known female factors responsible for infertility are ovulatory disorders (polycystic ovary syndrome, hypothalamic amenorrhea, and premature ovarian insufficiency), tubal pathology due to pelvic inflammatory disease or endometriosis, pelvic adhesions, endocrine disorders, and other tubal/structural problems [10]. Furthermore, some common factors for both males and females include age (>35 for females and >40 for males), metabolic disorders, stress, substance abuse, cigarette smoking, excessive alcohol consumption, exposure to radiation and environmental toxin exposure [4,11,12,13]. Male infertility factors have diverse pathophysiological mechanisms, including varicocele-induced testicular hyperthermia and oxidative stress, urogenital abnormalities, genetic causes (including chromosomal aneuploidies, Y-chromosome microdeletions, etc.), endocrinopathies, cryptorchidism with resultant spermatogenic failure, obstructive azoospermia, environmental exposures, idiopathic causes and lifestyle-related risks [14,15,16].

Recent advances have significantly improved our understanding of the mechanisms underlying male infertility, emphasizing the key roles of sperm DNA fragmentation, epigenetic abnormalities, mitochondrial impairment, alterations in seminal microbiomes, and exposure to environmental endocrine disruptors [17,18,19,20]. Novel molecular signatures, including aberrant DNA methylation patterns, dysregulated protamine expression ratios, altered metabolomic profiles, and defective CatSper function, have been identified as potential mechanisms of male infertility [17,21,22]. These insights are progressively transforming the diagnostic and therapeutic approaches of male infertility.

Some multicenter studies reported that nearly 50% of all the causes of infertility are female-related, whereas male factors are responsible for 20 to 30% cases of infertility, and about 20 to 30% cases are due to common factors of both sexes [23,24]. A study done by Aggarwal et al., which included only systematic reviews and meta-analyses, observed that there is a wide range of 20 to 70% of a presence of male factors in all infertility cases [24]. Nonetheless, this broad range of reported prevalence of male factors may not reflect the true prevalence of all parts of the world due to methodological inconsistencies in diagnostic criteria, inclusion bias, heterogeneity in data collection, and sociocultural constraints. However, evidence consistently indicates that the male factors contribute substantially to the etiology in affected couples [24,25].

Rationale for Narrative Review Approach:

Given the multifactorial nature of male infertility and the rapid expansion of research spanning metabolomics, environmental health, molecular diagnostics, and clinical management, a narrative review approach was chosen. This format allows the integration of diverse and emerging evidence, provides clinical context, and identifies knowledge gaps and future research priorities that may not be captured through narrowly focused systematic reviews.

**Figure 1:**
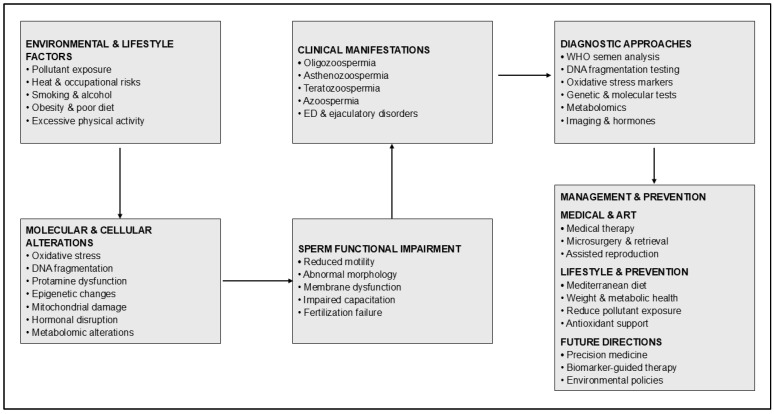
Multifactorial determinants of male infertility illustrating the progression from environmental and lifestyle exposures to molecular alterations, sperm dysfunction, clinical manifestations, diagnostic evaluation, and integrated management and prevention strategies.

This narrative review aims to provide a comprehensive understanding of the epidemiology and etiopathogenesis of male infertility, along with a critical appraisal of diagnostic modalities, including multi-omics and AI-enhanced assessment tools, and to evaluate the evidence-based treatment strategies, thereby highlighting critical knowledge gaps that warrant future research. This review integrates the latest scientific advances with established clinical principles to support evidence-based practice and encourage translational research focused on enhancing reproductive outcomes for couples with male factor infertility. The multifactorial determinants of male infertility are summarized in Figure 1.

## 2. Materials and Methods

### 2.1. Review Design and Reporting Guidelines

This narrative review was designed and reported in accordance with the Scale for the Assessment of Narrative Review Articles (SANRA) to enhance methodological rigor, transparency, and reproducibility [26]. The review also adheres to the Sex and Gender Equity in Research (SAGER) guidelines [27]. Although the primary focus of the review is male infertility from a biological sex perspective, distinctions between sex (biological attributes) and gender (sociocultural roles and identities) are acknowledged where relevant to infertility assessment and management.

### 2.2. Data Sources and Literature Search Strategy

This review is based on an extensive literature search conducted across PubMed/MEDLINE, Saudi Digital Library (SDL), Google Scholar, Psychological Information Database (PsycINFO), and other major databases from inception to December 2025. Reference lists of relevant articles were also manually screened to identify additional pertinent studies.

### 2.3. Search Terms

The search strategy utilized Medical Subject Headings (MeSH) terms and relevant keywords in various combinations using Boolean operators (AND/OR). The primary search terms included

- “Male infertility” OR “Male factor infertility”;
- “Spermatogenesis” OR “Sperm quality”;
- “Diagnosis” OR “Diagnostic techniques”;
- “Treatment” OR “Management” OR “Therapy”;
- “Epidemiology” OR “Prevalence”;
- “Assisted reproductive technology” OR “ART”.

### 2.4. Inclusion and Exclusion Criteria

The inclusion criteria were as follows:

- Peer-reviewed articles published in the English language.
- Studies reporting epidemiological data, etiology, diagnostic approaches, or treatment outcomes.
- Meta-analyses, systematic reviews, randomized controlled trials, observational studies, and case series.
- Publications from 2000 to 2025 (with older landmark studies included where appropriate).

The exclusion criteria were as follows:

- Non-English publications.
- Conference abstracts without full-text availability.
- Studies that lack adequate methodological detail.
- Duplicate publications.

### 2.5. Study Selection and Data Extraction

Titles and abstracts were initially screened for relevance, followed by full-text reviews of eligible studies. Data extraction focused on epidemiology patterns, underlying etiological mechanisms, diagnostic methods, treatment modalities, and emerging trends.

### 2.6. Quality Assessment

Given the narrative design, formal risk-of-bias tools were not systematically applied. However, emphasis was placed on high-quality evidence, prioritizing systematic reviews, meta-analyses, and well-designed clinical studies.

## 3. Main Text

Male infertility is defined as the inability of a male partner to induce pregnancy in a fertile female partner.

### 3.1. Epidemiology of Male Infertility: Global Prevalence and Emerging Trends

#### 3.1.1. Global Prevalence

Accurately determining the global prevalence of male infertility remains a complex task complicated by methodological complexity, heterogeneous diagnostic criteria, variable healthcare access, associated cultural stigma, and inconsistent reporting across epidemiological studies [28,29]. Male infertility is not properly reported in countries where there is the existence of a patriarchal social system [16,24]. For instance, in India, women are the ones mostly blamed for infertility. Furthermore, most of the time, to maintain masculinity and domination, men do not prefer to go for fertility evaluation [7,8]. Male infertility is considered a sensitive subject in many societies of the world, so in a population-based survey, most infertile couples may not agree to participate, leading to selection bias [9,16].

Epidemiologically, primary male infertility accounts for approximately 65–70% of male infertility cases, while secondary infertility represents 30–35% [30,31]. Emerging evidence from large population-based studies has provided precise estimates of male infertility prevalence across different regions of the world. In Middle Eastern populations, investigations from the Jeddah region of Saudi Arabia reported that male infertility was found among 65.6% of infertile couples, with 48% of semen abnormalities showing oligo-terato-asthenozoospermia in combination, whereas 52% had single-factor abnormality (teratozoospermia and oligozoospermia being common) [28]. A retrospective study conducted by Öztekin Ü et al. in the Anatolian region of Türkiye found that male-only factors contributed to 45.6% of the total infertility cases [32]. A meta-analysis conducted by Agarwal et al. reported that male factors contribute to infertility in approximately 20 to 70% of infertile couples, with the highest prevalence being observed in African and Eastern European males [24]. From 1990 to 2019, the global prevalence of male infertility increased by approximately 76.9%, reflecting a 19% increase since 1990. East Asia demonstrated the highest prevalence, whereas Western Sub-Saharan Africa exhibited the greatest relative increase. At the national level, China, India, and Indonesia accounted for the greatest number of cases, while Cameroon and Mauritania demonstrated the highest age-standardized rates [33]. Despite the wide range of variation in reporting male infertility rates by different authors, it is evident that male infertility prevalence is on the rise worldwide [25].

#### 3.1.2. Emerging Trends

One of the most important debates hovers around the concerns emanating from the documented declines observed in sperm concentration and total sperm counts across the globe. In a systematic review and meta-regression conducted by Levine et al., 2017, a substantial reduction in sperm concentration and total sperm counts was reported between 1973 and 2011, reflecting a 50–60% decline among unselected men by fertility group from Western populations [34]. Another meta-analysis done recently by Levine et al. in the year 2023 reported a significant decline in sperm counts among unselected men from other continents in addition to the Western populations [35]. These findings indicate a global decline that is persisting in the 21st century at an even faster rate, underscoring the importance of identifying the etiological factors and implementing measures to curb them.

The observed temporal trends have been attributed to multiple etiological factors, including exposure to environmental endocrine disruptors (pesticides, bisphenol A, dioxins, etc.), adverse lifestyle practices (obesity, sedentary habits, and high intake of processed foods), workplace-related hazards and heritable epigenetic modifications [36,37]. Recent evidence has implicated that microplastic exposure may contribute to detrimental effects on male reproductive health. Microplastics have been identified in human semen samples and have been associated with impaired sperm motility and morphology [38,39].

#### 3.1.3. Methodological Heterogeneity in Prevalence Estimates

Comparisons across studies should be interpreted cautiously due to methodological variability, including

- Differences in definitions and diagnostic criteria, e.g., infertility duration, WHO semen thresholds, and classification of male factor infertility.
- Study populations and sampling methods (clinic-based vs. population-based), introducing selection bias.
- Laboratory variability, including semen analysis techniques, quality control, and abstinence periods.
- Cultural and reporting factors influencing healthcare-seeking behavior and disclosure.
- Environmental and temporal factors including regional exposures, seasonal variation, and secular trends.

Implication: True prevalence is difficult to determine precisely; standardized population-based studies using consistent WHO criteria are needed. Despite heterogeneity, male factor infertility remains a major global contributor.

### 3.2. Classification and Causes of Male Infertility (Male Infertility Factors)

Male infertility is categorized into primary and secondary types based on prior pregnancy outcomes. Primary male infertility applies to men who have never achieved a pregnancy with their partner despite engaging in regular unprotected sexual intercourse. Secondary male infertility refers to men who have successfully achieved at least one pregnancy in the past but are unable to conceive currently [40].

Primary infertility is more commonly associated with congenital and genetic factors such as chromosomal abnormalities (e.g., Klinefelter Syndrome and Y chromosome microdeletions), cryptorchidism, and other developmental disorders. In contrast, secondary infertility is more linked to acquired conditions such as infections, trauma, varicocele, and lifestyle factors. This distinction has important prognostic and therapeutic implications. Regional variations exist, with secondary infertility more prevalent in areas where reproductive tract infections are common. Men with secondary infertility often benefit from treatment of reversible causes and lifestyle modification, whereas primary infertility may reflect irreversible genetic or developmental abnormalities requiring assisted reproductive techniques [41].

Male infertility causes are generally classified into coital infertility and azoospermia. Coital infertility is attributed to structural abnormalities (penile deformities), erectile dysfunction (ED), premature ejaculation, retrograde ejaculation, and anejaculation. The causes of azoospermia are classified into obstructive and non-obstructive [42]. Numerous studies worldwide have demonstrated that male factor infertility accounts for approximately 20 to 30% of the overall infertility burden [43]. However, male infertility is often underdiagnosed, as males are usually neglected while evaluating couple’s infertility [44]. Male infertility is an underacknowledged entity in the scientific field as well as in socioeconomic considerations [29]. Frequently, the cause of the male factor is not considered, and couples directly undergo assisted reproduction.

Another more comprehensive classification includes

**Figure 2:**
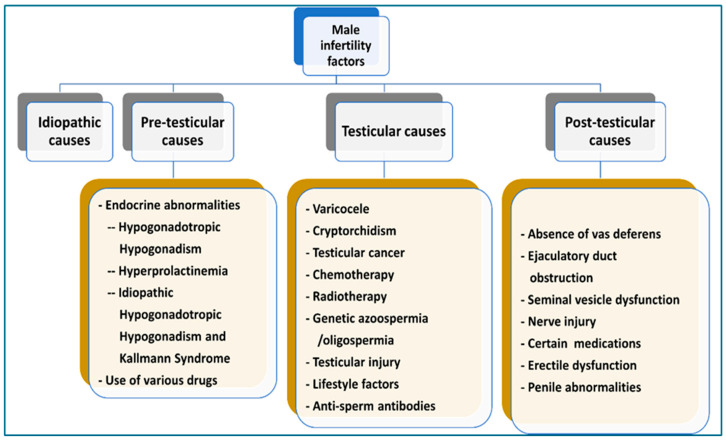
Male infertility factors.

- **Pre-testicular causes:** endocrine abnormalities and use of various drugs.
- **Testicular causes:** varicocele, cryptorchidism, testicular malignancies, exposure to chemotherapy or radiotherapy, genetic azoospermia/oligospermia, testicular trauma, and lifestyle factors.
- **Post-testicular causes:** absence of vas deferens, ejaculatory duct obstruction, seminal vesicle dysfunction nerve injury and use of certain medications [45] (Figure 2).

Despite advancements, the cause of male factor infertility remains unknown in 30–40% of cases despite thorough evaluation, and such cases are labeled as idiopathic male infertility. These cases have shown a growing association with underlying gene mutations, epigenetic alterations, and oxidative stress-driven damage to sperm DNA. Some of the critical male infertility factors are briefly discussed in this narrative review.

#### 3.2.1. Idiopathic Causes

Idiopathic male infertility constitutes nearly 30–40% of cases, in which no definitive cause can be identified despite thorough clinical and laboratory assessment [46]. Bracke et al. (2017) highlighted several potential contributors, including gene mutations, epigenetic dysregulation, post-translational protein modifications, alterations in the sperm proteome, and increased sperm DNA fragmentation [47]. The growing body of molecular evidence underscores the need for incorporating advanced diagnostic techniques to identify hidden abnormalities in otherwise unexplained infertility cases.

Multi-omics research has recently provided critical insights into the molecular basis of idiopathic infertility. Multi-omics investigations including genomic, transcriptomic, proteomic, and metabolomic analyses have delineated several pathways, including epigenetic dysregulation, protamine imbalance, mitochondrial dysfunction, CatSper channel deficiency and sperm DNA fragmentation (SDF). A detailed discussion of these mechanistic pathways and their clinical implications is provided in Section 3.3.5.

These emerging molecular insights have made it possible to explain many previously unexplained cases of male infertility, grouping them into specific mechanistic categories with potential clinical solutions.

#### 3.2.2. Varicocele

Varicocele refers to the abnormal dilatation of the veins of the spermatic cord and represents 19 to 41% cases of male infertility [48]. It is the most common and treatable cause of male infertility [49]. The pathogenesis of spermatogenic dysfunction in varicocele patients is multifactorial and includes the following:

1. **Testicular hyperthermia:** Impaired blood flow and venous stasis elevate testicular temperature by 2.5 °C, thereby causing damage to sperm DNA, apoptosis and hormonal imbalance, which ultimately disrupts temperature-sensitive spermatogenesis [50].
2. **Oxidative stress:** Hypoxia and impaired venous drainage associated with varicocele promote excessive ROS production, resulting in lipid peroxidation and damage to proteins and nucleic acids within testicular tissue [49].
3. **Hormonal dysregulation:** Varicocele adversely affects the semen quality and impairs Leydig cell dysfunction, leading to decreased testosterone and dihydrotestosterone (DHT) concentrations and compensatory elevations in the levels of follicle-stimulating hormone (FSH) and luteinizing hormone (LH) due to a lack of negative feedback on the pituitary gland [51].
4. **Reflux of adrenal and renal metabolites:** Animal studies have demonstrated the role of reflux of potentially toxic metabolites into the testicular circulation, which may lead to testicular damage and impaired spermatogenesis [52].

#### 3.2.3. Genetic and Epigenetic Causes

Genetic causes contribute to approximately 15% of male infertility cases, which can impair reproductive hormone balance, spermatogenesis, and sperm quality parameters. Nearly 2000 genes are involved in spermatogenesis, and males with azoospermia exhibit the greatest burden of genetic abnormalities [53]. Notable genetic causes include the following:

- **Chromosomal anomalies**: Klinefelter Syndrome (47XXY), Y Chromosome microdeletions, and partial deletion of azoospermia factors are the critical genetic causes for male infertility [54]. Other disorders include the following:
  **47,XYY Syndrome:** Men with this karyotype have normal external genitalia and may present with normal sperm counts or azoospermia. Fertility outcomes vary, with some men achieving natural conception while others require ART [55].
  **46,XX Male Syndrome (De la Chapelle Syndrome):** These individuals have a male phenotype but present with azoospermia due to the absence of Y chromosome genes essential for spermatogenesis. It results from translocation of the SRY gene to the X chromosome or an autosome [55].

- **Gene mutations**: Mutations in cystic fibrosis transmembrane conductance regulator (CFTR) lead to congenital bilateral absence of the vas deferens, presenting as obstructive azoospermia with normal spermatogenesis [53,56]. Additionally, mutations in the androgen receptor (AR) gene have also been implicated in male infertility [57,58].
- **Structural chromosomal rearrangements**: Translocations such as Robertsonian translocation 45,XY,t(13;14)(q10;q10) as well as unique reciprocal translocation t(6;12)(q23;q24.3) [59,60] and autosomal inversions [61,62] have also been associated with azoospermia/oligospermia in males.
- **Deafness-Infertility Syndrome (DIS):** This is a rare autosomal recessive disorder characterized by sensorineural hearing loss and male infertility due to defective sperm motility. The mutations caused include the CATSPER2 gene mutation, which interferes with the calcium channel function in sperm flagella [63].
- **Kartagener Syndrome:** This is a rare autosomal recessive disorder characterized by the triad of chronic sinusitis, bronchiectasis, and situs inversus, with male infertility resulting from impaired sperm motility due to absent or defective dynein arms in the sperm flagella [64].
- **Young Syndrome:** This is a rare inherited syndrome featuring reduced fertility due to azoospermia and chronic sinopulmonary infections, though the underlying genetic basis remains unclear [65].
- **Disorders of Sex Development (DSDs):** This broad category encompasses a spectrum of conditions with atypical development of chromosomal, gonadal, or anatomical sex. DSD affecting male fertility includes partial androgen insensitivity syndrome (PAIS), mixed gonadal dysgenesis, 5-alpha reductase deficiency and ovotesticular DSD. Such patients usually present with ambiguous genitalia and impaired spermatogenesis, often requiring multidisciplinary management [66].

#### 3.2.4. Endocrinopathies

Endocrinopathies are an important detectable and often correctable cause of male infertility but contribute to only 1–2% of the causes of male infertility. Important endocrine disorders associated with infertility are as follows.

- **Hypergonadotropic Hypogonadism (primary hypogonadism)**

This is caused by primary testicular dysfunction that may be induced by testicular injury due to trauma, tumor, chemotherapy or radiation, as well as may be seen in Klinefelter Syndrome. It typically presents with reduced serum testosterone and elevated LH and FSH levels [67].

- **Hypogonadotropic Hypogonadism (secondary hypogonadism)**

Hypothalamic or pituitary defects lead to inadequate Leydig cell stimulation, which reduces the testosterone levels. The FSH and LH levels in these patients are low or low–normal [67]. The etiologies include congenital conditions, including Kallmann Syndrome, idiopathic Hypogonadotropic Hypogonadism, as well as disorders linked to various genetic mutations [68]. Acquired causes include hypothalamic and pituitary disorders or lesions, infiltrative diseases, head trauma, radiotherapy and androgen/anabolic steroid use [69]. Appropriate hormonal therapy can often restore fertility in secondary hypogonadism, whereas those with primary hypogonadism usually do not respond to such treatments [67].

- **Hyperprolactinemia**

Elevated prolactin suppresses pulsatile release of gonadotrophins and impairs spermatogenesis, thereby leading to male infertility. Contributing causes include prolactinomas and use of prolactin-elevating medications, such as antipsychotics and metoclopramide.

Thyroid dysfunction, particularly hypothyroidism, has been associated with impaired semen quality [70].

- **Congenital Adrenal Hyperplasia (CAH)**

CAH, an autosomal recessive disorder most commonly caused by 21-hydroxylase deficiency, can cause male infertility through multiple mechanisms that include suppression of gonadotropins by elevated adrenal androgens, testicular adrenal rest tumors (TARTs) causing mechanical obstruction of seminal ducts and testicular damage, and inadequate hormonal control. Management includes glucocorticoid replacement therapy and surgical resection of large TARTs if present. Many men with CAH can achieve fertility after appropriate treatment [71].

#### 3.2.5. Cryptorchidism

Cryptorchidism, or undescended testes, is a well-documented cause of impaired spermatogenesis. The pathophysiological mechanisms involved are as follows:

- Germ cell loss: Undescended testes are exposed to abnormally high temperatures, which leads to secondary germ cell depletion and later infertility [72].
- Sertoli cell and Leydig cell dysfunction: Impaired testosterone production and altered hormonal milieu have been observed in cryptorchidism [73].
- Increased malignancy risk: There is 4–6-fold increased risk of developing testicular cancer and the risk may persist despite orchiopexy [74].

Cryptorchidism is associated with decreased sperm concentration and compromised sperm quality Men with cryptorchidism tend to have reduced sperm counts and poor quality of sperm [14]. The risk of infertility is significantly higher in bilateral cases [75] where the incidence of azoospermia is proportionally increased [76]. The infertility risk is proportional to the time the testicles remain undescended, as well as to the distance from the normal location of testis [14]. It is recommended to have surgical correction, preferably before 1 year of age in such cases to minimize long-term fertility risks.

**Testicular Dysgenesis Syndrome (TDS)**

Testicular Dysgenesis Syndrome (TDS) is a developmental disorder concept that links several male reproductive abnormalities including cryptorchidism, hypospadias, testicular cancer, and impaired spermatogenesis. The pathogenesis is thought to involve disruption of Sertoli and Leydig cell development during fetal life, influenced by genetic susceptibility and environmental endocrine-disrupting chemicals.

TDS has significant implications for male infertility, and men with a history of cryptorchidism or hypospadias should be counseled about increased risks of infertility and testicular cancer. These abnormalities may result in impaired spermatogenesis, reduced semen quality, and increased risk of subfertility or infertility in adulthood. The TDS framework emphasizes the need for early diagnosis and timely management for cryptorchidism, as well as surveillance for testicular malignancy in individuals at increased risk [77].

#### 3.2.6. Lifestyle and Environmental Factors

Multiple modifiable lifestyle and environmental factors have been associated with a reduction in male fertility. Those factors include obesity, smoking, alcohol consumption and other substance abuses, genital tract infections, poor nutritional status, psychological stress, chronic exposure to excessive heat, and toxic environmental exposures [78,79,80]. These are outlined below as follows:

- **Obesity and Metabolic Syndrome:** Several pathogenic mechanisms have been implicated, including hormonal dysregulation due to increased aromatase activity, which increases estradiol production, thereby suppressing the hypothalamic–pituitary–gonadal axis [81]; oxidative stress with reactive oxygen species production that damages the sperm membrane and sperm DNA [82]; scrotal hyperthermia resulting from increased scrotal adiposity that ultimately affects heat-sensitive spermatogenesis [83]; and erectile dysfunction due to vascular and neurogenic involvement [84]. Multiple studies have demonstrated an association between obesity and impaired semen parameters, including decreased sperm concentration, altered motility, and morphological abnormalities [85,86,87]. Weight loss interventions have been shown to improve semen parameters and hormonal profiles in obese men [88].
- **Tobacco and substance use:** Cigarette smoking is associated with reduced sperm counts, and decreased sperm motility and the effect is more pronounced in moderate to heavy smokers [89]. Smoking has a negative impact on sperm DNA integrity, thereby increasing sperm DNA fragmentation; the effects may be reversible upon cessation of smoking [90]. Regular marijuana smoking (>once per week) decreased total sperm counts by 29%, whereas its use along with other recreational drugs further decreased counts by 55% [91]. Anabolic–androgenic steroid abuse can cause severe but preventable causes of hypogonadism in males [92].
- **Physical activity and male fertility: an inverted U-shaped relationship:** Moderate exercise benefits male fertility, whereas excessive physical activity can paradoxically impair spermatogenesis—a critical but underrecognized phenomenon.
  **Benefits of moderate exercise include**
  - Improved testosterone levels;
  - Reduced oxidative stress;
  - Enhanced insulin sensitivity;
  - Improved blood flow to genital area and improved sperm quality [93].
  **Adverse effects of excessive training are as follows:**
  Prolonged high-intensity exercise, particularly among endurance athletes (e.g., marathon runners, cyclists, and triathletes), has been associated with impaired spermatogenesis.
  **Proposed mechanisms include**
  ▪ Hypothalamic–pituitary–gonadal axis suppression with decreased testosterone production;
  ▪ Increased oxidative stress leading to sperm DNA damage;
  ▪ Scrotal hyperthermia impairs spermatogenesis.
  **Anabolic steroid misuse**, sometimes associated with bodybuilding, can cause severe Hypogonadotropic Hypogonadism and marked impairment of spermatogenesis [94].
  These findings support an inverted U-shaped relationship between physical activity and male fertility, in which moderate exercise is beneficial, while excessive training may be detrimental.

- **Environmental endocrine disruptors:** Environmental pollutants are increasingly recognized as major contributors to male infertility. In addition to altering conventional semen parameters, these toxicants induce molecular and cellular changes that impair fertilization potential and may affect offspring health. Commonly environmental endocrine disruptors include the following:
  **Phthalates:** These are widely used plasticizers that exert anti-androgenic effects, disrupt steroidogenesis, alter gene expression and epigenetic processes, and increase oxidative stress and mitochondrial dysfunction. Exposure is associated with reduced sperm count, motility, and increased DNA fragmentation [95].
  **Bisphenol A (BPA):** BPA exerts anti-androgenic and pro-estrogenic effects through receptor-mediated signaling and oxidative stress pathways. It disrupts calcium signaling and causes epigenetic modifications via DNA methylation. BPA exposure has been linked to impaired sperm concentration, reduced motility, and increased DNA damage [96,97].
  **Persistent organic pollutants (POPs):** Organochlorine pesticides like DDT, polychlorinated biphenyls, dioxins, etc., accumulate in reproductive tissues, impairing spermatogenesis and promoting inflammatory and oxidative stress responses [98].
  **Microplastics:** Exposure to microplastics compromises the blood–testis barrier, disrupts the hormonal balance and induces oxidative stress-mediated sperm DNA damage [99].
  **Heavy Metals: arsenic (As), lead (Pb), cadmium (Cd), mercury (Hg):** They induce oxidative stress, cause hormonal disruption by inactivating steroidogenic enzymes, lower the protamine/histone ratio, induce epigenetic alterations and disrupt spermatogenesis, leading to reduced sperm count, reduced motility, abnormal morphology, and elevated DNA fragmentation [100].

- **Molecular alterations induced by environmental endocrine** disruptors: Environmental toxicants impair male fertility through mechanisms extending beyond routine semen analysis:
  1. **Sperm nuclear basic proteins and DNA integrity:**
    Protamine 1 and 2 replace histones during spermiogenesis, thereby compacting and protecting DNA from oxidative damage. Environmental pollutants disrupt protamine expression and function, resulting in incomplete histone-to-protamine transition, an abnormal P1/P2 ratio, defective chromatin condensation, increased susceptibility to oxidative damage, and impaired fertilization potential [100,101].
    Recent evidence suggests that in highly polluted environments, dysfunctional protamines may paradoxically contribute to oxidative DNA damage rather than protecting the genome, highlighting their role in environmentally mediated reproductive toxicity.
  2. **Epigenetic modifications:**
    Pollutant exposure modifies DNA methylation patterns, histone marks, and sperm non-coding RNAs, including miRNAs and piRNAs, potentially affecting spermatogenesis, embryo development and transgenerational health outcomes [100].
  3. **Mitochondrial dysfunction:**
    Toxicants damage sperm mitochondria, reducing ATP production and increasing reactive oxygen species, thereby impairing motility and sperm function [102].
  4. **Membrane integrity and receptor function:**
    Environmental contaminants may alter sperm membrane composition and fertilization-related proteins, disrupting capacitation, acrosome reaction, and sperm–oocyte binding [103].
  These environmental exposures contribute to male infertility not only by altering semen parameters but also through molecular and epigenetic disruptions that impair fertilization and embryo development.

- **Sperm as a sentinel biomarker of environmental health**
  Emerging evidence indicates that semen quality is not merely a reproductive parameter but a sentinel indicator of overall male health and environmental quality. Spermatozoa are increasingly regarded as sensitive indicators of the interaction between environmental exposure and human health. Spermatogenesis is highly vulnerable to toxic insults, particularly pollutant-induced disruption of sperm nuclear protein–DNA interactions leading to sperm DNA damage. Declining sperm quality has been associated with industrial pollution, pesticide exposure, and urban environmental contamination. These observations support the use of sperm molecular integrity as an early biomarker of environmental and reproductive risk [100].

- **Occupational and heat exposures**
  Occupations involving prolonged sitting (taxi drivers and office workers) or heat exposure (steel welders, ceramic oven operators, bakers, and foundry workers) correlate with impaired semen parameters [104]. Frequent use of laptop computers and excessive exposure to saunas may lead to reversible impairment of spermatogenesis [105,106].

Collectively, these lifestyle factors influence hormonal balance, oxidative stress levels, and spermatogenesis, underscoring the importance of holistic management in male infertility.

#### 3.2.7. Infectious Causes of Male Infertility

**Viral infections that can cause male infertility are as follows:**

- Mumps orchitis: Up to 30% of post-pubertal males with mumps orchitis may develop subfertility or infertility due to testicular atrophy, the underlying pathogenesis being orchitis-induced testicular atrophy and resultant azoospermia. Vaccination is highly effective in reducing the incidence of mumps [107].
- Other viral infections, including HIV, HPV, cytomegalovirus, and herpes simplex virus, may impair sperm parameters through direct viral effects and immune-mediated mechanisms [108].

**Bacterial infections that can cause male infertility are as follows:**

- Chlamydia trachomatis: It is the most common sexually transmitted bacterial infection and may lead to epididymitis, prostatitis, and urethritis. No vaccine is available for this infection; therefore, screening of at-risk populations as well as early diagnosis and treatment is recommended [109].
- Neisseria gonorrhoeae: This causes epididymo-orchitis, urethritis, and prostatitis [110].
- Mycoplasma and ureaplasma species: These are associated with anti-sperm antibodies, sperm DNA damage and decreased sperm motility. Pathogenetic mechanisms include direct damage as well as immunologically mediated damage to sperm [111].

**Genital tuberculosis:**

- It remains a critical cause of obstructive azoospermia and chronic inflammation of the reproductive organs in the endemic regions. Mycobacterium tuberculosis causes epididymo-orchitis with epididymal obstruction, as well as granulomatous inflammation of vas deferens, prostate and seminal vesicles. Management includes anti-tubercular therapy and surgical correction when necessary [112].

#### 3.2.8. Differentiation of Male Genital Tract Inflammatory Conditions

**Urethritis**

- It presents with urethral discharge and dysuria and may adversely affect semen quality [113].

**Prostatitis**

- Prostatitis is classified into acute bacterial, chronic bacterial, chronic prostatitis/chronic pelvic pain syndrome (CP/CPPS), and asymptomatic inflammatory prostatitis. CP/CPPS has been associated with impaired sperm parameters and sexual dysfunction. Proposed mechanisms include inflammatory cytokine release, oxidative stress, DNA fragmentation, and altered prostatic secretions [114].

**Epididymitis**

- Chronic inflammation may lead to ductal obstruction and impaired sperm transport. A sterile form of epididymitis may occur in **Behçet’testis**, a multisystem vasculitis that can involve the testis, epididymis, and associated ducts, potentially impairing fertility. Recognition is important because management requires immunosuppressive therapy rather than antibiotics [115].

**Male Accessory Gland Infection (MAGI)**

MAGI encompass infections of the prostate, seminal vesicles, and bulbourethral glands, affecting 2–18% of infertile men. MAGI may impair fertility through excessive reactive oxygen species production, inflammatory cytokine release, altered accessory gland secretions, sperm DNA damage, and obstructive lesions of the seminal tract. WHO criteria consider abnormalities in semen parameters together with clinical and microbiological findings for the diagnosis of MAGI. Evaluation of accessory gland function markers involves the following:

- Fructose: seminal vesicle marker; absent or low levels indicate seminal vesicle agenesis or ejaculatory duct obstruction.
- Neutral α-glucosidase: epididymal marker; reduced levels suggest epididymal dysfunction or obstruction.
- Zinc: prostatic marker; low levels indicate prostatic dysfunction.
- Citric acid and acid phosphatase: prostatic marker.

Combined evaluation of these markers helps localize the site of obstruction or dysfunction.

Management typically includes targeted antibiotic therapy, anti-inflammatory agents, and antioxidants [116].

#### 3.2.9. Post-Testicular Causes

**Types of ejaculatory dysfunction are as follows:**

- **Retrograde ejaculation:** Retrograde ejaculation is characterized by impairment in the forward expulsion of the seminal fluid, often resulting in infertility and emotional or psychological distress. Causes include diabetes mellitus, medications (α-blockers, antipsychotics, and antihypertensives), retroperitoneal lymph node dissection (RPLND), spinal cord trauma, and transurethral resection of prostate (TURP) and bladder neck surgery [117].
- **Retroperitoneal lymph node dissection (RPLND):** Retroperitoneal lymph node dissection (RPLND), performed in testicular cancer management, may damage sympathetic nerves responsible for emission and bladder neck closure. Nerve-sparing techniques have reduced this risk; however, injury may result in retrograde ejaculation or anejaculation [117].
- **Spinal cord injury (SCI):** Spinal cord injury disrupts sympathetic pathways controlling emission and bladder neck closure, resulting in ejaculatory dysfunction and retrograde ejaculation [117].
- **Anejaculation**: Anejaculation refers to the absence of seminal emission during orgasm [117].

**Types of structural anomalies are as follows:**

- **Hypospadias:** Mild distal hypospadias rarely affects fertility, whereas severe proximal forms may impair semen deposition and are sometimes associated with abnormal semen parameters and hormonal disturbances [118].
- **Peyronie’s Disease:** It is characterized by penile curvature due to fibrotic plaque in the tunica albuginea. Infertility may result from mechanical difficulty with intercourse, associated erectile dysfunction, and psychological distress [119].
- **Müllerian duct cysts:** Large cysts may compress the ejaculatory ducts, resulting in obstructive azoospermia [120].
- **Hematospermia**: Hematospermia is usually benign but may be associated with infection, inflammation, or structural abnormalities. Anxiety and associated sexual dysfunction may indirectly affect fertility [121].

**Postorgasmic Illness Syndrome (POIS):**

POIS is a rare disorder marked by debilitating physical and cognitive symptoms occurring immediately after ejaculation and lasting for 2–7 days. Symptoms include profound fatigue, cognitive difficulties, flu-like features (myalgia and low grade fever), and anxiety or mood changes. The proposed mechanisms include autoimmune/allergic reaction, cytokine-mediated inflammatory response and opioid receptor dysfunction.

Diagnosis is clinical, based on the consistent temporal relationship to ejaculation. POIS may significantly impair quality of life and reduce sexual activity, thereby affecting fertility attempts. Management strategies such as hyposensitization therapy and symptomatic pharmacologic treatments have shown variable success [122].

### 3.3. Diagnostic Methods for Male Infertility Factors: From Conventional Assessment to Multi-Omics Integration

Recently, several advancements have been made in the diagnosis of male infertility factors. This narrative review focuses on male infertility factors and evaluation. However, it is imperative to note that the female factors should be evaluated simultaneously for the complete infertility assessment for a successful conception in a couple.

**Classification of Diagnostic Tests by Clinical Application**

To improve clarity and guide clinicians in test selection, diagnostic approaches can be categorized according to their current clinical utility:

**Tier 1: routine clinical evaluation**

(Recommended for all infertile men undergoing infertility assessment)

- Comprehensive history and physical examination.
- Semen analysis (WHO 2021 criteria) [123].
- Hormonal evaluation (testosterone, FSH, and LH ± prolactin).
- Scrotal ultrasound.
- Post-ejaculatory urinalysis when retrograde ejaculation is suspected.

**Tier 2: specialized tests (indicated cases)**

(Performed based on clinical findings or specific indications)

- Genetic testing (karyotype, Y-chromosome microdeletions, and CFTR mutations).
- Sperm DNA fragmentation testing in unexplained infertility, recurrent pregnancy loss, or ART failure.
- Anti-sperm antibody testing in cases of sperm agglutination or post-vasectomy reversal.
- Transrectal ultrasound for suspected ejaculatory duct obstruction.
- Testicular biopsy to differentiate obstructive from non-obstructive azoospermia.
- MRI for complex anatomical abnormalities.

**Tier 3: advanced and emerging techniques**

(Available in specialized centers; clinical utility continues to evolve)

- Oxidative stress assessment (ROS, ORP).
- Chromatin maturity and integrity tests.
- Advanced sperm selection techniques (IMSI and microfluidic devices).
- Metabolomic and epigenetic profiling.
- AI-assisted semen analysis and specialized sperm function assays.

**Clinical Application**

Tier 1 tests form the foundation of evaluation and are indicated in all cases, Tier 2 investigations are guided by specific clinical indications, and Tier 3 tools are reserved for selected cases and research or specialized settings.

#### 3.3.1. Clinical Evaluation

A meticulous and complete clinical history forms the cornerstone of infertility assessment. Key components include the following:

- **Reproductive history**: duration of infertility, past pregnancy, sexual history (erectile and ejaculatory dysfunction, frequency of masturbation, etc.).
- **Medical history**: previous surgery information, past illnesses and present comorbid health conditions and current medications.
- **Personal history**: smoking, substance abuse and alcohol consumption and occupational history to assess exposure to toxins.
- **Family history**: genetic and reproductive disorders [124,125].

Physical examination is the essential tool for infertility assessment among men. Initial evaluation should include a general physical examination with assessment of height, weight, and body mass index (BMI), followed by evaluation of sexual development [124]. For the sexual development assessment, clinicians must look for gynecomastia and signs of androgen deficiencies like diminished body hair distribution. A thorough genital examination should be performed to identify structural abnormalities, Peyronie plaques, epispadias, hypospadias, and evidence of sexually transmitted diseases. Two instruments, namely the Prader Orchidometer and Pencil Probe Doppler Stethoscope, are essentially used for the physical assessment of subfertility and infertility among males [126]. The Prader Orchidometer was invented in 1966 to measure testicular volumes through twelve numbered (1 to 25 mL) ovoid-shaped beads, which are readily available methods for estimating spermatogenesis in adolescents and adult males [127]. Normally, testicular volume must be a minimum of 15 mL and testis length should be a minimum of 4 cm. Even though varicocele can be diagnosed by physical examination, subfertile and infertile males are further examined through Pencil Probe Doppler Stethoscope in a standing/upright position and the instrument to be kept on the upper part of the scrotum. Varicocele of the testis and blood reflux is indicated by hearing a venous rush during the Valsalva maneuver [126].

Emerging anthropometric indicators are as follows:

**Anogenital distance (AGD):** Anogenital distance (AGD) is the distance from the center of the anus to the base of the penis, determined during fetal life under androgen influence. It serves as a biomarker of prenatal androgen exposure and testicular function. Shorter AGD has been associated with reduced testosterone levels, lower sperm counts, and poor semen quality. AGD is a promising clinical marker for evaluating male reproductive potential and identifying men at risk of infertility [128].

**2D:4D digit ratio:** The ratio of the index finger (2D) to ring finger (4D) length reflects prenatal androgen exposure. A lower ratio suggests higher androgen exposure in utero. Research indicates that lower ratios correlate with higher sperm counts and better semen parameters, whereas higher ratios have been associated with reduced semen quality [129].

#### 3.3.2. Imaging Techniques

Imaging techniques used for diagnosing male infertility are ultrasound techniques, MRI and invasive imaging techniques:

- **Scrotal ultrasound** is the preferred initial choice and includes transverse, longitudinal and color Doppler ultrasound studies. It is safe, non-invasive and readily available [130]. This assessment facilitates the identification of testicular abnormalities, determination of testicular volume, and detection of peritesticular structural changes, including varicoceles [131].
- **Transrectal ultrasound** is the investigation of choice for evaluating the vas deferens, seminal vesicles and prostate. This technique is particularly useful for diagnosing obstructive azoospermia [130].
- **Color Doppler of penis** is used to evaluate for erectile dysfunction [130].
- **MRI** may be superior to ultrasound to visualize accessory sex structures [131].
- **Invasive imaging techniques** include testicular venography and embolization for diagnosing varicoceles [132] and vasography for evaluation of the vas deferens and ejaculatory duct [130]. Invasive imaging techniques are not the preferred modalities because of their invasive nature and the risk of associated infections [130].

**Advanced imaging modalities are as follows:**

- **Testicular thermography:** It is non-invasive infrared imaging technique for detecting temperature variations in the scrotal contents. Its main application lies in detecting varicocele, but it can be used in other conditions like testicular torsion and inflammatory conditions [133].
- **Testicular scintigraphy:** It is a nuclear medicine technique using Technetium-99m pertechnetate to assess testicular perfusion. Primary indication is in acute scrotum patients to differentiate between testicular torsion and epididymitis. It has a limited role in routine infertility evaluation [134].
- **High-definition magnetic resonance imaging (MRI):** It provides superior soft tissue resolution and has multiplanar capability when compared to ultrasound. Applications in male infertility include detection of seminal tract abnormalities, testicular volume estimation and differentiating between obstructive and non-obstructive azoospermia (NOA) [135].

**Advanced techniques for TESE outcome prediction are as follows:**

- **Apparent diffusion coefficient (ADC) mapping:** Diffusion-weighted MRI assesses water molecule diffusion in testicular tissues. Higher ADC values have been associated with successful sperm retrieval in non-obstructive azoospermia. This non-invasive technique may help select appropriate candidates for TESE and guide surgical planning [136].
- **Ultrasound measurement of seminiferous tubule diameter:** High-frequency ultrasound is used to measure the diameter of seminiferous tubules. In some studies, a diameter ≥250 μm has been associated with successful sperm retrieval during micro-TESE in men with non-obstructive azoospermia. This technique is promising due to its accessibility and cost-effectiveness [137].
- **Transrectal ultrasound (TRUS):** Transrectal ultrasound (TRUS) is essential for evaluating post-testicular pathology, including ejaculatory duct obstruction, midline prostatic cysts, seminal vesicle abnormalities, and prostatic disease. It is particularly valuable because many identified conditions are potentially correctable [138].

TRUS-guided seminal vesicle aspiration is both a diagnostic and therapeutic procedure. It enables the analysis of seminal vesicle fluid in suspected obstruction or infection and allows decompression of cysts or abscesses [139]. It is particularly useful in the diagnosis of partial ejaculatory duct obstruction [140] and may facilitate sperm retrieval in obstructive cases [141].

#### 3.3.3. Semen Analysis: The Cornerstone of Male Fertility Assessment

The semen parameter analysis is the mainstay evaluation of the male infertility factor [142]. Due to the high degree of variability in semen analysis, it is advisable to collect two independent samples at least a week apart after the abstinence of three days of sexual intercourse or night wet dreams. The WHO has published guidelines for the collection and analysis of semen through standard methodology. In general, semen collection in a laboratory setting is recommended. However, home collection also can be done. But it must be collected in a proper container, stored at room temperature, and should reach the laboratory within one hour after collection for analysis. After receipt, the specimen containers are placed in an incubator at 37 °C for 5 min to access liquefaction. Between 30 and 60 min, assessment of liquefaction, semen appearance, semen volume measurement, semen PH assessment, sperm motility, vitality, and concentration are to be done. After 4 h, straining and assessing smears for sperm morphology is to be performed [123].

**Evolution of WHO Laboratory Manual for Semen Analysis**

The WHO has published six editions of the laboratory manual for examination and processing of human semen (1980, 1987, 1992, 1999, 2010, and 2021), reflecting advances in reproductive science and evolving concepts of male fertility [143].

Key changes across editions are as follows:

- Early editions (1980–1999) focused on standardizing basic semen analysis procedures, later incorporating optional tests, quality control measures, and statistical approaches to improve laboratory accuracy.
- The 2010 (5th) edition marked a major shift toward evidence-based reference values derived from fertile men whose partners conceived within 12 months. Lower reference limits based on the 5th percentile were introduced.
- The 2021 (6th) edition further refined methodologies, expanded reference datasets, strengthened quality assurance practices, and emphasized clinical interpretation of semen parameters rather than rigid thresholds [143].

Current WHO 2021 reference values (5th percentile, 95% CI) are summarized in Table 1 [144].

The WHO manual emphasizes the need to record both morphologically normal spermatozoa as well as spermatozoa with abnormalities of the head, midpiece and principal piece. Three indices to record these abnormalities are the teratozoospermia index, the multiple anomalies index and sperm deformity index (SDI) [123].

The WHO manual standardizes terminology for semen abnormalities to ensure consistent reporting and clinical communication. Standardized nomenclature for semen abnormalities according to WHO 2021 guidelines are as follows:

- Oligozoospermia: sperm count less than 16 million/mL.
- Azoospermia: complete absence of spermatozoa in ejaculate.
- Teratozoospermia: normal morphological forms <4%.
- Asthenozoospermia: reduced sperm motility, defined as progressive motility less than 30% and/or total motility below 42%.
- Oligoteratozoospermia: having less than 4% normal morphology and less than 16 million sperm per ml.
- Asthenoteratozoospermia: having less than 42% of all motile sperm and less than 4% of sperm with normal morphology.
- Oligoasthenoteratozoospermia (OAT syndrome): all three parameters abnormal, with less than 16 million sperm/mL, less than 42% total motility, and less than 4% of normal morphology

Standardized nomenclature facilitates uniform reporting, enhances communication between laboratories and clinicians, and improves clinical decision-making [145].

Critical points to be considered are as follows:

- **Repeated semen analysis:** Given the significant intra-individual variability, it is recommended to do at least two analyses separated by 2–4 weeks [146].
- **Standardization:** Significant inter-laboratory variability as well as the highly subjective nature of manual evaluation has necessitated the need for standardized, automated systems [147].
- **Careful clinical interpretation:** WHO reference values are statistical benchmarks rather than distinct indicators of fertility or infertility, as men with values below these ranges may remain fertile and those within or above them may still experience infertility [148].
- **Limitations:** Conventional semen analysis does not assess sperm DNA integrity, functional status of spermatozoa, or its fertilization capacity [149].

**Sperm morphology assessment is performed as follows:**

- **Kruger’s Strict Criteria (Tygerberg Criteria):**
  Developed by Dr. Thinus Kruger, this stringent morphology assessment evaluates sperm head shape, acrosomal size, midpiece alignment, and tail structure using strict dimensional standards. A morphology of ≥4% normal forms is considered within the lower reference limit. Strict morphology has prognostic value in natural conception and assisted reproduction, particularly in predicting fertilization outcomes [150].

- **Computer-aided sperm analysis (CASA):**
  CASA provides an objective, automated assessment of sperm motility and kinematics. Key parameters include the following:
  - VAP (Average Path Velocity)—velocity along a smoothed trajectory.
  - VSL (Straight-Line Velocity)—velocity from start to endpoint.
  - VCL (Curvilinear Velocity)—velocity along the actual path.
  - ALH (Amplitude of Lateral Head Displacement)—reflects flagellar vigor.
  - BCF (Beat Cross Frequency)—frequency of head movement across the trajectory.

CASA improves objectivity and reduces interobserver variability while providing detailed motion analysis. Limitations include high equipment cost and the need for technical expertise [151].

**Sperm Vitality Assessment**

Vitality testing distinguishes immotile but viable sperm from nonviable sperm and is recommended when total motility is below 40%:

- **Eosin–nigrosin staining (supravital staining)**
  Eosin dye can penetrate the damaged membranes of dead sperm but is excluded by intact membranes of live sperm. Live sperm appear unstained, whereas dead sperm appear pink/red. Advantages include a permanent preparation along with simultaneous morphology assessment.

- **Hypo-osmotic swelling test (HOST):**
  Viable sperm with intact membranes undergo swelling in a hypo-osmotic solution, producing characteristic tail coiling. Non-viable sperm show no tail changes. The normal threshold is ≥58% of spermatozoa with tail swelling.
  Clinical applications of these approaches are as follows:
  - Differentiation of asthenozoospermia from necrozoospermia.
  - Selection of viable sperm prior to ICSI [152].

**Semen Hyperviscosity**

Semen that does not liquefy within 60 min at room temperature or forms threads >2 cm when dropped from a pipette is defined as hyperviscous. Causes include dysfunction of prostatic and seminal vesicles and infectious disorders. Semen hyperviscosity impairs sperm motility and interferes with sperm transport in female reproductive tract.

Management includes antibiotics for infection, anti-inflammatory agents, mucolytic enzymes and adequate hydration. Persistent hyperviscosity may require assisted reproduction techniques [153].

**Seminal Plasma Biochemistry**

Beyond standard semen parameters, biochemical analysis of seminal plasma provides insight into accessory gland function and helps localize reproductive tract pathology. Key markers such as fructose, neutral α-glucosidase, and zinc are discussed in the section on accessory gland evaluation (MAGI section).

**Seminal Plasma Metallomics**

The seminal plasma metallome, comprising essential trace elements and potential toxic metals, plays a critical role in spermatogenesis and sperm function. Essential elements such as zinc, selenium, magnesium, calcium, copper, and manganese contribute to antioxidant defense, membrane stability, sperm motility, capacitation, and DNA integrity. Imbalances or deficiencies may impair sperm quality.

In contrast, toxic heavy metals including lead, cadmium, and mercury can accumulate in reproductive tissues, inducing oxidative stress, DNA damage, and impaired spermatogenesis. Occupational and environmental exposures are increasingly recognized contributors to male infertility.

Metallomic profiling, using techniques such as inductively coupled plasma mass spectrometry (ICP-MS), may help identify deficiencies or toxic exposures and is an emerging research tool in idiopathic infertility [154].

**Split Ejaculation Sampling**

Ejaculate fractionation involves collecting semen in sequential portions, most commonly three fractions:

- **First fraction:** rich in spermatozoa and prostatic secretions, typically showing higher sperm concentration and motility.
- **Subsequent fractions:** contain increasing contributions from seminal vesicle secretions and lower sperm density.

Clinical applications of these fractions are as follows:

- The first fraction may provide higher-quality sperm for assisted reproductive techniques (ARTs).
- This is useful in men with borderline semen parameters.
- Although not routinely performed, it represents a simple method to optimize sperm selection for assisted reproduction [155].

**Protocol for Distinguishing Azoospermia from Cryptozoospermia**

When no spermatozoa are observed on initial microscopic examination, centrifugation of the semen sample is required to distinguish true azoospermia from cryptozoospermia.

The centrifugation protocol is as follows:

- Transfer the entire ejaculate to a conical centrifuge tube.
- Centrifuge at ≥3000 *g* for 15 min.
- Carefully remove the supernatant, leaving the pellet undisturbed.
- Resuspend and examine the entire pellet microscopically.

The classification of this protocol is as follows:

- Azoospermia: no spermatozoa found in fresh sample or centrifuged pellet;
- Cryptozoospermia: no sperm observed in the fresh sample, but rare spermatozoa detected after centrifugation.

The clinical significance is as follows:

- Cryptozoospermia indicates residual spermatogenesis and a more favorable reproductive potential.
- Even rare sperm can be used for ICSI.
- Cryopreservation is recommended whenever spermatozoa are identified [156].

#### 3.3.4. Advanced Sperm Function Tests and Biomarkers

A range of promising biomarkers for both diagnosis and prognosis has been elucidated, including the sperm DNA fragmentation index, anti-sperm antibodies, and oxidative stress markers in seminal fluid [157].

**Sperm DNA fragmentation index (DFI):** Sperm DNA fragmentation reflects changes in the bases of DNA, single- and double-strand DNA breaks, formation of DNA adducts, and pyrimidine dimers [158]. It occurs significantly in infertile men when compared to fertile men [159]. Measurement techniques include the following:

**TUNEL (terminal deoxynucleotidyl transferase dUTP nick end labeling):** detects DNA strand breaks through flow cytometry or fluorescent microscopy and is the most common test used [160].

**Sperm chromatin structure assay (SCSA):** flow cytometry-based; detects fragmentation of DNA as well as chromatin structure [160].

**Sperm chromatin dispersion (SCD or Halo test):** indirectly measures the sperm DNA fragmentation after lysis of sperm and acid denaturation by utilizing fluorescent microscopy [160].

**Comet assay**: an electrophoresis-based technique to estimate sperm DNA strand breaks; requires a fresh sample but can be performed on a small number of sperm [161].

Recent advances include automated AI-integrated models evaluating live sperm morphology, which offer high accuracy and decrease assessment time, leading to enhanced outcomes for fertility treatments [162]. AI-integrated semen analysis with the integration of clinical and imaging data has significantly enhanced the stratification and identification of varicocele patients likely to benefit from early surgical intervention [163].

**Immunological Testing for Anti-sperm Antibodies (ASA)**

Anti-sperm antibodies arise from an autoimmune response against sperm antigens and can impair sperm motility, cervical mucus penetration, and fertilization.

Indications for ASA testing include

- History of testicular trauma, torsion, or surgery;
- Testicular cancer;
- Asthenozoospermia;
- Sperm agglutination;
- Vasectomy reversal;
- Genital tract infections.

**Diagnostic Tests**
**Mixed Antiglobulin Reaction (MAR) Test**

This is a direct test detecting antibodies bound to sperm using latex particles coated with anti-human immunoglobulin. Binding of particles to motile sperm indicates ASA. The elements of this procedure are as follows:

- >50% bound motile sperm suggests clinically significant antibody presence.
- Separate tests for IgG and IgA classes

The advantages of this approach are that it is simple, rapid, and uses fresh semen and that it is cost-effective.

**Immunobead Test (IBT)**

This test uses antibody-coated beads applied to washed motile sperm to detect ASA and identify binding sites. The elements of this procedure are as follows:

- Binding to the sperm head is associated with impaired fertilization.
- IgA antibodies are particularly associated with reduced fertilization potential.
- It is more sensitive and site-specific than MAR testing.
- **Indirect Tests (Less Commonly Used)**

Indirect tests are done if the sample is oligozoospermic or asthenozoospermic in case of obstructive azoospermia, or if a sample cannot be tested [164].

**Oxidative Stress Assessment**
**Oxidation-Reduction Potential (ORP) Test**

This test measures the balance between oxidants and reductants in semen. The MiOXSYS system provides a rapid electrochemical assessment, with values above approximately 1.36 mV/10^6^ sperm/mL associated with oxidative stress. Elevated ORP correlates with DNA fragmentation, poor motility, and ART outcomes.

**The clinical utility** of this test is the identification of oxidative stress in idiopathic infertility and guidance of antioxidant therapy. Limitations include reduced reliability in highly viscous or azoospermic samples [165].

**Endtz test or Peroxidase Staining for Leukocyte Detection**

This distinguishes peroxidase-positive leukocytes from germ cells using peroxidase enzyme activity. Stained leukocytes appear brown. The WHO threshold for leukocytospermia is ≥1 × 10^6^ peroxidase-positive cells/mL [165].

**DNA Integrity Assessment**
**Sperm Chromatin Structure Assay (SCSA) and High DNA Stainability (HDS) Index**

The SCSA test uses flow cytometry to assess the DNA fragmentation index (DFI) and high DNA stainability (HDS). The high DNA stainability (HDS) index reflects immature spermatozoa with incomplete chromatin condensation. While elevated HDS has been associated with impaired fertility in some studies, its independent clinical predictive value remains uncertain [166].

**Specialized Sperm Function Tests**
**Mouse Oocyte Activation Test (MOAT)**

This test evaluates the sperm’s ability to activate the oocyte after membrane fusion, reflecting phospholipase C zeta (PLCζ) function. It is primarily used in cases of repeated fertilization failure after ICSI and globozoospermia to guide artificial oocyte activation strategies [167].

**Hamster Egg Penetration Test (HEPT)/Sperm Penetration Assay (SPA)**

This test assesses sperm’s capacitation, acrosome reaction, and fusion capability using zona-free hamster oocytes. Although it can detect subtle functional defects, its clinical use has declined due to ICSI availability, ethical concerns, and high interlaboratory variability [168].

**Acrosome Reaction Assessment**

This is assessed using fluorescent staining (FITC-PSA and FITC-PNA), flow cytometry and immunocytochemistry [169]. Abnormal acrosome reaction responses may indicate fertilization defects [170].

**Transmission Electron Microscopy (TEM) for Ultrastructural Sperm Analysis**

TEM provides ultra-high-resolution imaging of sperm ultrastructure, essential for diagnosing specific structural defects not visible by light microscopy.

**The indications of this approach are as follows:**

- Asthenozoospermia;
- Suspected flagellar, acrosomal and mitochondrial abnormalities;
- Multiple ART failures and research on sperm pathology.

**The ultrastructural defects identified by TEM are as follows:**

- Flagellar defects like primary ciliary dyskinesia, dysplasia of fibrous sheath (DFS syndrome) and multiple morphological flagellar anomalies.
- Acrosomal defects like globozoospermia and macrozoospermia.
- Nuclear defects like chromatin condensation abnormalities and nuclear membrane defects.
- Midpiece defects such as mitochondrial abnormalities.

Clinical significance includes the diagnosis of specific genetic syndromes and guiding genetic counseling and treatment options, explanation of severe asthenozoospermia with normal vitality, and prediction of ICSI outcomes. Limitations include the requirement of specialized equipment and expertise and that it is expensive and time-consuming [171].

#### 3.3.5. Multi-Omics Profiling: The Future of Precision Diagnostics

Advanced multi-omics approaches integrate genomic, epigenomic, transcriptomic, proteomic, and metabolomic data to elucidate molecular phenotypes underlying infertility [172].

**Genomics:** Whole-exome sequencing and whole-genome sequencing have identified pathogenic variants in spermatogenesis genes that lead to spermatogenic failure and infertility [173]. Changes in the mitochondrial genome may lead to impaired oxidative phosphorylation and reduced ATP production, thereby leading to abnormal sperm parameters [174]. Mutations affecting *CATSPER* genes lead to dysfunctional CatSper calcium channels, thereby interfering with sperm hyperactivation required for penetration of the egg coat and successful fertilization. This may represent one of the most frequent underlying causes of unexplained male infertility [175].

**Epigenomics:** The principal epigenetic mechanisms that play a critical role in spermatogenesis and early embryonic development include DNA methylation,of histone modifications and ncRNAs. Environmental exposures can disrupt these epigenetic mechanisms, thereby leading to male infertility [19]. In a review done by Rotondo et al. in 2021, it was observed that aberrant DNA methylation patterns involving various genes like MEST, H19 and MTHFR were linked with male infertility [20]. DNA methylation abnormalities have been reported in men with oligoasthenoteratozoospermia and oligozoospermia [176,177].

In a review done by Oliva R 2006, protamine alterations, including changes in the P1/P2 ratio and alteration in the phosphorylation and alkylation of protamines, were observed to be a cause of male infertility [178]. The role of RNAs in the maturation of spermatozoa is well-documented, and higher RNA has been observed in poor-quality semen [179]. Increased expression of circular RNAs has been documented in asthenozoospermic patients [180].

**Proteomics:** Several biomarkers, including SPA17, ANXA2, and SERPINA5 [181]; testis-specific proteins and germ-cell-enriched heat-shock proteins [182]; and dysregulated expression of proteins involved in oxidative phosphorylation and glycolysis, have been identified in infertile men [183].

**Sperm DNA fragmentation (SDF)**: Sperm DNA fragmentation (SDF) has emerged as an important molecular marker in the evaluation of male infertility. High SDF is associated with higher rates of abortions in IVF and ICSI patients. High levels of sperm DNA fragmentation have been reported in patients with varicocele and in infertile men with normal semen analysis [90,184]. These findings highlight the limitations of routine semen analysis and the clinical value of advanced DNA integrity testing.

**Metabolomics in Male Infertility: Identifying Root Causes**

Metabolomics, which involves the comprehensive analysis of small-molecule metabolites, offers an advanced approach for understanding male infertility and identifying novel biomarkers. Integrated metabolome–microbiome analyses have identified dysbiosis patterns in idiopathic infertility [18].

Metabolomic analyses can be performed on seminal plasma, sperm cells, urine, and plasma. Among these, the seminal plasma metabolome reflects metabolic activity of the testes, epididymis, prostate, and seminal vesicles, while the sperm metabolome provides insight into sperm functional competence. Techniques such as mass spectrometry and nuclear magnetic resonance spectroscopy enable high-throughput metabolite profiling [185].

- **Metabolic Alterations Associated with Male Infertility**
  Studies have identified characteristic metabolic disturbances in infertile men:
  **Energy metabolism dysfunction:** Altered glycolytic metabolites (elevated lactate and pyruvate) and reduced Krebs cycle intermediates suggest impaired mitochondrial function and reduced ATP production, contributing to poor sperm motility [185].
  **Amino acid metabolism abnormalities:** Reduced levels of carnitine and acetyl-carnitine, essential for mitochondrial energy transport, and altered amino acids (e.g., glycine, serine, and proline) have been associated with impaired spermatogenesis and idiopathic infertility [185].
  **Lipid metabolism disruption:** Altered phospholipid composition, increased oxysterols, and reduced omega-3 fatty acids (DHA) affect sperm membrane integrity, motility, and fertilization capacity [185].
  **Oxidative stress imbalance:** Reduced glutathione (GSH) and elevated oxidized glutathione (GSSG) indicate oxidative stress, a key mechanism underlying sperm DNA damage and reduced sperm function [186].
  **Nucleotide metabolism alterations:** Altered purine and pyrimidine metabolism are associated with DNA fragmentation and impaired chromatin integrity [187].
  **Hormonal and signaling metabolites:** Altered steroid metabolites may reflect disruptions in the local hormonal microenvironment, affecting spermatogenesis.

- **Clinical Applications of Metabolomics:**
  Metabolomics offers several clinical advantages:
  **Identifying root causes:** Metabolomics reveals underlying pathology, such as oxidative stress, mitochondrial dysfunction, inflammation, or endocrine imbalance.
  **Diagnostic biomarkers:** Distinct metabolite profiles may help differentiate fertile from infertile men and characterize subtypes such as asthenozoospermia or idiopathic infertility.
  **Personalized treatment:** Metabolomics may guide targeted, therapy including antioxidant therapy, carnitine supplementation, or lifestyle modifications.
  **Prediction of ART outcomes:** Metabolic markers may help predict fertilization potential and assisted reproduction success [188].
**Future perspectives:**
  Metabolomics provides deeper insights into the molecular basis of male infertility by identifying specific metabolic dysfunctions that support precision diagnostics and personalized treatment. Integration of metabolomics with genomics, proteomics, and microbiome analyses may further enhance the understanding of idiopathic infertility [185].
  Metabolomic profiling complements genetic and proteomic analyses, providing functional insight into sperm physiology and environmental influences.

**Transcriptomics:** Sperm RNA signatures, especially microRNAs (miRNAs) and Piwi-interacting RNAs (piRNAs), are emerging as valuable biomarkers for assessing semen quality and anticipating pregnancy results [189].

Although encouraging, the adoption of multi-omics diagnostics requires validation in prospective studies, rigorous standardization across labs, cost reduction, and evidence demonstrating superior predictive performance relative to conventional assessments.

**Novel Biomarkers for Non-Obstructive Azoospermia**

Recent studies have identified novel biomarkers for non-obstructive azoospermia:

- Anoikis-related genes that may be diagnostic biomarkers for NOA, offering views into the underlying molecular mechanisms and therapeutic targets [190].
- Whole-exome sequencing (WES) and seminal/serum biomarkers like anti-Müllerian hormone and testis-expressed sequence 101 protein can help predict micro-TESE outcomes, thereby reducing the likelihood of procedure failure [191].
- Serum NEAT1 and miR-34a are potential diagnostic biomarkers for male infertility and may assist in guiding individualized treatment strategies [192].

#### 3.3.6. Reproductive Hormonal Assay

The reproductive hormonal imbalance may lead to infertility among males, and the affected hormones are LH, FSH, prolactin, and testosterone. FSH stimulates sperm production, LH helps in the secretion of testosterone, and prolactin is one of the crucial hormones regulating male fertility by regulating LH and FSH secretion [193]. Hormonal assessment is suggested in the following situations, namely, sperm concentration <10 million/mL, semen volume <1 mL, history of erectile dysfunction, and patients with clinical features suggestive of hypogonadism [126].

Hormonal assays are performed through a fully automated analyzer that uses electrochemiluminescence technology for immunoassay analysis, and these methods are most widely used. However, mass spectrometry-based methods have also been widely employed and have proved their sensitivity, specificity, and reliability [194]:

- **Total testosterone levels:** Diagnosis of male hypogonadism requires two separate early morning serum samples, and values below 300 ng/dL are considered diagnostic [195]. Even though hormonal testing can be done at any time, it is recommended to withdraw the blood from 7 to 9 AM especially in men below 45 years of age, as they tend to have the highest testosterone level during this time [196].
- **LH and FSH levels:** In primary hypogonadism, LH and FSH levels are typically elevated, accompanied by reduced serum testosterone. In contrast, secondary hypogonadism is characterized by reduced testosterone levels and low or low–normal FSH and LH levels [67].
- Additional hormonal evaluation may include prolactin, estradiol and TSH.
- **Hormonal ratios with predictive utility include the** anti-Müllerian hormone to testosterone ratio (AMH/tT) and the inhibin B to AMH ratio (INHB/AMH), which have proven to be of predictive value in idiopathic non-obstructive azospermic patients undergoing sperm retrieval procedures [197].

#### 3.3.7. Testicular FNAC and Biopsy

Testicular biopsy is performed under anesthesia, and it is advised for infertile males due to idiopathic reasons and azoospermia. Numerous debates around testicular biopsy have persisted over the past decades, as it usually does not describe the exact cause of infertility. In most cases, it will not alter the therapeutic options. However, these procedures can be beneficial among males with obstructive oligospermia. Testicular biopsy specimens can be obtained by three procedures: open biopsy, microsurgical aspiration, and percutaneous puncture (true-cut or fine needle aspiration) [198,199].

The accuracy of testicular FNAC to diagnose male infertility approaches 91.9%, whereas it is 100% accurate in diagnosing normal spermatogenic activity in obstructive azoospermia. Unilateral testicular FNAC or biopsy is generally sufficient for establishing diagnosis in most cases of male infertility [200].

Histopathological evaluation in infertile men may reveal the following:

- **Normal spermatogenesis:** suggests obstructive azoospermia.
- **Hypospermatogenesis:** reduced germ cell numbers.
- **Germ cell arrest:** spermatogenesis halts at specific stages and carries poor prognosis for spermatozoid recovery.
- **Sertoli cell-only syndrome:** characterized by the complete absence of germ cells within the seminiferous tubules [201].

**Histological Evaluation and Johnsen’s Scoring System**

Testicular histology is commonly assessed using the Johnsen Scoring System, a standardized 10-point scale that evaluates spermatogenic activity within seminiferous tubules. A score of 10 indicates complete spermatogenesis with mature germ cells, whereas a score of 1 indicates total absence of germ cells. This method provides a practical correlation between semen analysis findings and testicular biopsy results [202].

At least 50–100 seminiferous tubules are examined, and a score is assigned to each tubule. The mean score is calculated and the range of scores observed is reported [203] (Table 2).

In the clinical interpretation of this system, scores of 9–10 indicate normal or near-normal spermatogenesis and suggest obstructive azoospermia when semen lacks spermatozoa, whereas a score of 1 indicates testicular atrophy/sclerosis, and likelihood of sperm retrieval is low. Higher Johnsen scores are generally associated with an increased likelihood of successful sperm retrieval, although micro-TESE may occasionally identify sperm even in cases with low histological scores due to focal spermatogenesis [204].

Limitations include interobserver variability, sampling error, and focal spermatogenesis, which may allow sperm retrieval despite low scores [202].

#### 3.3.8. Urine Analysis

Urine analysis is considered as one of the cost-effective screening methods to detect serum FSH levels, as low FSH in urine is directly associated with low serum FSH [205]. The diagnostic accuracy of urinary FSH measurement shows 100% sensitivity for identifying corresponding serum FSH levels, although its sensitivity for detecting low sperm counts is comparatively lower at 58% [206]. Postejaculate urinalysis (PEU) is a widely utilized test for evaluating male infertility, particularly for confirming retrograde ejaculation in men with low seminal volume. The presence of >10–15 sperm per high-power field (HPF) in post-ejaculatory urine is considered diagnostic of retrograde ejaculation [207,208].

### 3.4. Treatment of Male Infertility

**Figure 3:**
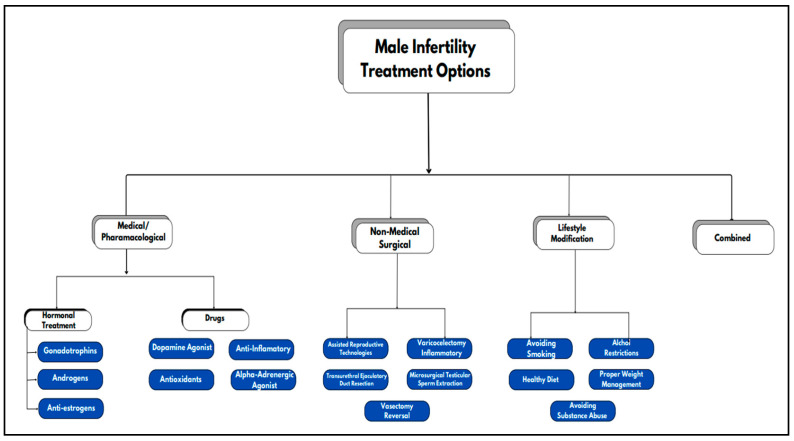
Overview of different treatment modalities of male infertility.

Identification of the underlying etiological factors allows targeted therapeutic interventions and improves the likelihood of successful reproductive outcomes. Globally, 17.5% of adults are experiencing infertility, as reported by the WHO, and male factors contribute significantly to this prevalence rate [209]. Timely and appropriate management of male infertility may significantly enhance the psychological well-being and reproductive outcomes at the level of the individual, couple, and broader community [210,211]. The primary objective of male infertility treatment is to optimize male reproductive potential to a level where they can make their female partners achieve a clinical pregnancy. However, male infertility treatment is a complex phenomenon due to a considerable proportion of unknown etiologies of male infertility [11,212]. This section describes the treatment modalities most commonly used for male infertility. The different treatment modalities for male infertility are summarized in Figure 3.

#### 3.4.1. Hormonal Treatment

Hormonal therapy remains a mainstay for treating idiopathic male infertility and hypogonadism. Such patients are typically managed with gonadotrophins substitutes such as human chorionic gonadotrophins (hCGs). Examples of hormonal treatments are as follows:

- **Human chorionic gonadotrophins (hCGs):** In general, hCG is self-administered through subcutaneous route by the patients (1500 to 3000 IU/biweekly) for 6 months to two years [213]. Patients should be adequately counseled about the possible adverse effects, including hyperglycemia, gynecomastia, and depressive symptoms. Pharmacologically, human chorionic gonadotrophin (hCG) mimics LH activity, recombinant FSH provides FSH activity, and human menopausal gonadotropin (hMG) combines both FSH and LH actions. Pulsatile GnRH therapy is especially indicated in patients having gonadotropin deficiency due to hypothalamic disorders. The drawbacks of this therapy are the formation of anti-GnRH antibodies in some cases and the need to wear a pulsatile pump [214].
- **Combination therapy with gonadotropins**: This is used in cases of failure to achieve desired results with hCG monotherapy [215].
- **Anti-estrogens:** Anti-estrogenes, namely clomiphene citrate [216] and tamoxifen, are the commonly used estrogen receptor antagonists to treat male infertility [217]. It is important for males to maintain a normal testosterone to estradiol ratio (T:E), and an imbalance in the T:E ratio may decrease the secretion of LH and FSH, leading to decreased spermatogenesis.
- **Anastrozole:** This is an aromatase inhibitor used in infertile males with abnormal T:E ratios [218].

#### 3.4.2. Dopamine Agonists

The therapeutic benefits of dopamine agonists, particularly bromocriptine and cabergoline, have been extensively evaluated in the management of male infertility. Among these agents, cabergoline is generally preferred due to its superior efficacy and better tolerability [219].

#### 3.4.3. Antioxidants

Reactive oxygen species (ROS) and free radical-mediated oxygen stress may damage the spermatozoa membrane, leading to defective sperm function and resulting in abnormal semen parameters. Antioxidant supplementation has shown benefits among infertile and subfertile males by diminishing ROS production and thereby leading to enhanced semen quality [220,221,222].

**Evidence-Based Antioxidant Therapy**

Oxidative stress plays a central role in idiopathic male infertility by impairing sperm function, membrane integrity, and DNA stability. Several antioxidants and micronutrients have been investigated for their potential to improve semen parameters and reproductive outcomes.

**Coenzyme Q10 (Ubiquinone)**

This is a powerful lipid-soluble antioxidant and an essential component of the mitochondrial electron transport chain; it is essential for energy production and protects against oxidative stress. The dosage is 200–400 mg daily for at least 3–6 months. Evidence of its use includes clinical studies and meta-analyses that reported improvements in sperm concentration, motility, and morphology, along with reductions in oxidative stress and DNA fragmentation. Benefits appear most pronounced in men with idiopathic oligoasthenoteratozoospermia. Some studies suggest improved pregnancy outcomes, although evidence remains heterogeneous [223].

**Astaxanthin**

This is a lipid-soluble carotenoid with exceptionally high antioxidant potency. It also possesses anti-inflammatory and anti-apoptotic effects. It protects sperm membrane and DNA. The dosage is 12–16 mg daily for 3 months. Research suggests improvements in sperm parameters and reductions in lipid peroxidation. Preliminary data indicate potential benefits in fertility outcomes, though large controlled trials remain limited [224].

**Lycopene**

This is a carotenoid pigment (found in tomatoes); it is a potent singlet oxygen quencher and protects against lipid peroxidation. The dosage is 6–8 mg twice daily for 3 months. Supplementation has been associated with improvements in sperm concentration, motility, and oxidative stress markers. Some studies suggest reduced DNA damage and improved pregnancy [222].

**Zinc**

Zinc is an essential micronutrient for spermatogenesis, testosterone metabolism and antioxidant defense. It stabilizes sperm membranes and protects DNA. The dosage is 25–50 mg elemental zinc daily. Zinc deficiency is associated with impaired sperm quality. Supplementation may improve sperm count, motility, and testosterone levels, particularly when combined with other antioxidants. Excess intake should be avoided [222].

**Selenium**

It is a component of glutathione peroxidase; it protects against oxidative damage and is essential for sperm motility and morphology. The dosage is 200 µg/day. Low selenium levels are associated with impaired sperm motility and morphology. Combined supplementation with antioxidants (e.g., N-acetylcysteine) has shown improvement in semen parameters in clinical studies [222].

**Emerging Therapies**
**Probiotics and Gut Microbiota Modulation**

Emerging evidence suggests that the gut microbiota influences systemic inflammation, oxidative stress, and hormonal regulation. Dysbiosis may negatively impact spermatogenesis and sperm function. Probiotic supplementation has been associated with improvements in semen parameters and reductions in oxidative stress in preliminary studies; however, further research is needed to establish clinical recommendations [225].

**Polyamines (Spermine and Spermidine):**

Polyamines such as spermidine are involved in cellular growth, DNA stabilization, and regulation of oxidative stress. Experimental studies suggest a role in spermatogenesis and sperm maturation. While early findings indicate potential reproductive benefits, clinical evidence remains limited [226].

**Clinical considerations of the above are as follows:**

- Antioxidant therapy may benefit men with oxidative stress-related infertility.
- Combination therapy may provide synergistic effects.
- Evidence varies in the quality and magnitude of benefits.
- Supplements should complement, not replace, evaluation and treatment of underlying causes.

#### 3.4.4. Specific Pharmacological Agents for Idiopathic Male Infertility

Idiopathic male infertility is frequently associated with oxidative stress, mitochondrial dysfunction, and impaired spermatogenesis. Several pharmacological agents and supplements have been investigated to improve sperm function and reproductive outcomes.

**L-Carnitine and L-Acetyl-Carnitine**

Carnitines play a vital role in sperm energy metabolism by facilitating fatty acid transport into mitochondria for ATP production, which is essential for sperm motility. Their antioxidant properties may also protect spermatozoa from oxidative damage. Clinical studies and meta-analyses suggest improvements in sperm motility, concentration, and morphology, particularly in men with idiopathic infertility [227].

**Vitamin C (Ascorbic Acid)**

This is a water-soluble antioxidant in seminal plasma that neutralizes ROS and protects sperm DNA from oxidative damage. Supplementation (typically 500–1000 mg/day) has been associated with improvements in sperm count, motility, and DNA integrity. Some studies suggest enhanced ICSI outcomes and increased spontaneous pregnancies when combined with other antioxidants [228].

**Vitamin E (α-Tocopherol)**

This is a lipid-soluble antioxidant that protects the sperm membrane from lipid peroxidation and helps maintain membrane integrity. Supplementation (commonly 400 IU/day) has been associated with improvement in sperm motility and reduced DNA damage. Combined therapy with selenium or vitamin C may enhance antioxidant effects [228].

**Kallidinogenase**

Kallidinogenase has historically been used to improve testicular microcirculation through vasodilatory effects, with the aim of enhancing spermatogenesis. However, contemporary evidence supporting its clinical benefit is limited, and its routine use has declined.

**Kampo Medicine (Traditional Japanese Herbal Medicine)**

Common formulations for male infertility are as follows:

- Hochuekkito: for erectile dysfunction and male infertility.
- Hachimijiogan: for erectile dysfunction.
- Keishikaryukotuboreito: for erectile dysfunction.

These exert antioxidant, anti-inflammatory, and hormone-modulating effects and may improve testicular microcirculation [229]. Some Japanese studies report improvement in sperm concentration and motility; however, large randomized controlled trials are limited, and further research is required [230].

#### 3.4.5. Other Drugs

Some researchers reported the benefits of corticosteroids and nonsteroidal anti-inflammatory drugs (NSAIDs) to improve the fertility status among idiopathic male infertility factors. The administration of corticosteroids is particularly recommended in idiopathic oligozoospermic patients with genital inflammation [231]. However, an appropriate antibiotic regimen should be considered in the presence of genital tract infections [232,233].

#### 3.4.6. Management of Retrograde Ejaculation

**Medical management of retrograde ejaculation is outlined as follows:**

**α-Adrenergic agonists (first line):**

- Pseudoephedrine: 60–120 mg daily or prior to ejaculation attempt.
- They increase seminal output and promote antegrade ejaculation.
- Contraindications: hypertension and cardiovascular diseases.
- Side effects: elevated blood pressure, palpitations, insomnia, and anxiety [117].

**Tricyclic antidepressants:**

- Imipramine: most used; 25-50-100 mg daily or prior to ejaculation attempt.
- Mechanism: anticholinergic effects increase bladder neck resistance
- Success rates increased when combined with sympathomimetics [117].

**Sperm Retrieval from Urine (When Medical Management Fails)**

Pre-collection preparation includes a urine alkalinization protocol, as normal urine pH is toxic to sperm. The patient voids immediately before an ejaculation attempt. Ejaculation is attempted in a sterile container. Immediate post-ejaculatory urine collection into another sterile container is performed. Laboratory processing consists of centrifuging urine at 300× *g* for 10 min, removing the supernatant and resuspending the pellet in a warm wash medium. Sperm concentration, motility, and viability are assessed.

**Clinical outcomes are as follows:**

- Urine alkalinization improves sperm motility.
- Retrieved sperm may be used for IUI or IVF/ICSI.
- The Hotchkiss method: bladder catheterization followed by instillation of Ringer’s lactate prior to ejaculation may improve sperm recovery [117].
**Surgical management**
  Surgical management is reserved for anatomical causes like bladder neck reconstruction. However, ART is preferred in such cases [117].
**Alternative sperm retrieval**

If urine retrieval is unsuccessful, the following approaches can be used:

- Electroejaculation (EEJ) [234].
- Penile vibratory stimulation (PVS) [234].
- Testicular sperm extraction (TESE) as a last resort [117].

#### 3.4.7. Management of Erectile Dysfunction (ED) in Infertility

Erectile dysfunction means failure to achieve or maintain an erection sufficient for satisfactory sexual performance. It is common in men over 40 years of age and shows increasing prevalence worldwide. ED may impair semen deposition and contribute to coital infertility.

**Comprehensive diagnostic evaluation is performed as follows:**

**Clinical assessment:**

- Sexual history and medical history.
- Validated questionnaires: IIEF-5 (International Index of Erectile Function).
- Physical examination including genital, cardiovascular and neurological evaluation.

**Laboratory investigations:**

- Testosterone (total and free), LH, FSH, and prolactin.
- Fasting glucose, HbA1c and lipid profile.
- Thyroid function.

**Specialized diagnostic tests are as follows:**

**Nocturnal Penile Tumescence (NPT) testing:**

- NPT can be assessed using devices such as RigiScan, which measures penile rigidity and tumescence during sleep, or the stamp test, a simple home-based screening method.
- Differentiates psychogenic (normal nocturnal erections) from organic ED (absent or reduced nocturnal erections) [235].

**Penile Doppler ultrasound:**

- Assesses penile blood flow after intracavernosal vasodilator injection followed by measurement of peak systolic velocity (PSV) and end-diastolic velocity (EDV).
- Arterial insufficiency: PSV <25 cm/s.
- Venous leak: elevated EDV >5 cm/s [235].

**Cavernosometry and cavernosography:**

- Invasive tests for venous leak.
- Rarely performed; reserved for surgical candidates.

**Treatment Approaches**

**First-line therapies are as follows:**

**Phosphodiesterase-5 Inhibitors (PDE5i):**

- For example, sildenafil and tadalafil; enhance cavernosal smooth muscle relaxation and penile blood flow.
- Sildenafil (Viagra): 25–100 mg, 1 h before intercourse.
- Effective in the majority of patients.
- Contraindicated with nitrates [235].

**Lifestyle modifications:**

- Weight control and exercise.
- Smoking and alcohol cessation.
- Dietary management.

**Psychosexual counseling:**

- For psychogenic ED or relationship issues
- Couples therapy [235].

**Second-line therapies are as follows:**

**Intracavernosal injections (ICI):**

- Prostaglandin E1 alone or in combination (Trimix and QuadMix).
- Increases cavernosal cyclic AMP, which causes smooth muscle relaxation.
- Highly effective; risks include priapism, fibrosis, and pain.

**Intraurethral alprostadil (MUSE):**

- Medicated urethral system for erection. Alprostadil pellet inserted into urethra.
- Efficacy: 50–65%.
- Safe and less invasive than injections.

**Vacuum erection devices (VEDs):**

- Cylinder placed over penis, vacuum creates negative pressure, drawing blood into penis. Constriction ring is placed at base to maintain erection that lasts up to 30 min.
- Efficacy: 70–80%.
- Safe and effective, though patient satisfaction rates vary.

**Low-intensity extracorporeal shockwave therapy (LI-ESWT):**

- This therapy may improve penile vascular function in selected patients.
- Used in patients with severe ED not responding to PDE-5 inhibitors.
- Modest improvements reported in selected patients [235].

**Third-line (Surgical) therapies are as follows:**

**Penile prosthesis implantation:**

- Indicated when conservative treatments fail.
- Inflatable prosthesis: activated by squeezing pump in scrotum. Usually preferred by younger men and men with decreased penile sensations.
- Malleable prosthesis: can be manipulated into straight or bent position. Usually preferred in older men.
- High patient and partner satisfaction rates.

**Penile revascularization surgery:**

- Indicated in young men (<30 years) with isolated arterial insufficiency due to traumatic injury to pelvis or perineum. Bypass from inferior epigastric artery to dorsal penile artery.
- Rarely performed, and long-term outcomes show only minimal improvement.

**Venous ligation surgery:**

- Used for venous leak (veno-occlusive dysfunction). Involves embolization or ligating the penile veins. Not recommended, as long-term success is poor [235].

**Adjunctive treatments are as follows:**

**Testosterone replacement therapy (TRT):**

- Testosterone replacement therapy may improve libido and erectile function in hypogonadal men but is not a primary treatment for ED [235].

#### 3.4.8. Sperm Retrieval in Anejaculation and Severe Ejaculatory Dysfunction

**Penile Vibratory Stimulation (PVS)**

PVS is a non-invasive technique that induces ejaculation by activating the ejaculatory reflex arc through high-frequency vibratory stimulation of the glans penis.

Indications are as follows:

- Spinal cord injury (SCI) with an intact ejaculatory reflex arc (above T10).
- Idiopathic anejaculation.
- Multiple sclerosis and diabetes with autonomic neuropathy.

Advantages are as follows:

- Non-invasive, well tolerated and a first-line option.
- High success rate.

Disadvantages include risk of autonomic dysreflexia in SCI patients (injuries above T6) and lower sperm quality than normal ejaculation.

**Electroejaculation (EEJ)**

It involves electrical stimulation of pelvic nerves via a rectal probe to induce emission and ejaculation.

Indications are as follows:

- Spinal cord injury (when PVS fails).
- Refractory retrograde ejaculation.
- Severe neurological disorders.
- Psychogenic anejaculation (refractory cases).

It is highly effective in inducing ejaculation in SCI patients. Complications include autonomic dysreflexia in SCI above T6, rectal mucosal injury and pain.

**Recommendations are as follows:**

- PVS is preferred initial approach (non-invasive).
- EEJ is highly effective when PVS fails.
- Sperm cryopreservation is advisable for future cycles.
- ART often required due to suboptimal sperm parameters [234].

#### 3.4.9. Surgical Sperm Retrieval: Optimizing Techniques for Azoospermia

- **Obstructive azoospermia:** Percutaneous epididymal sperm aspiration (PESA), microsurgical epididymal sperm aspiration (MESA) and testicular sperm aspiration (TESA) give a high sperm retrieval rate in the range of 90–100% [236]. Vasoepididymostomy or vasovasostomy includes microsurgical reconstruction for epididymal or vasal obstruction and was able to obtain mean patency rate of 87% and mean pregnancy rate of 49% [237].
- **Non-obstructive azoospermia:** Microsurgical TESE is considered the gold standard, with 40–60% sperm retrieval rates [238]. Conventional TESE has 30–40% sperm retrieval rates, whereas TESA has a retrieval rate of only 20–30% [238]. Predictive biomarkers of successful sperm recovery include FSH, total testosterone, inhibin B and TEX101 [239]. Novel techniques include intraoperative optical coherence tomography for real-time tubule assessment; multiphoton microscopy (MPM) that specifically targets sperm-containing tubules; and the integration of artificial intelligence (AI) and machine learning (ML) technologies [238].
- **Salvage Options for Untreatable Azoospermia**
  When sperm retrieval fails in men with non-obstructive azoospermia, comprehensive counseling regarding alternative family-building options is essential.
**Donor Sperm Insemination/IVF with Donor Sperm (AID/IVF-D)**
**The indications are as follows:**
  - Non-obstructive azoospermia at multiple TESE attempts.
  - Multiple ICSI failures.
  - Single women seeking conception.
  - Genetic conditions precluding biological fatherhood.
  - Severe genetic risks to offspring.

Anonymous donors (sperm bank) are selected after proper screening. IUI is performed with donor sperm for the female partner with normal fertility and IVF with donor sperm if the female factor is present or IUI failed.

Advantages include high success rates, especially in ICSI failure patients, female partner retaining genetic a link to the child, and an established legal framework in most countries.

Challenges include psychological, social, legal and ethical issues [240].

**Adoption**

Adoption provides an alternative path to parenthood for couples who choose not to pursue donor gametes or further assisted reproduction. Counselling should include discussion of emotional, ethical, cultural, and legal considerations [40].

#### 3.4.10. Advanced Microsurgical Techniques

**Microsurgical Vasoepididymostomy (MVE)**

This involves microsurgical anastomosis between the vas deferens and epididymal tubule for epididymal obstruction. Indications include epididymal obstruction (congenital, post-infectious, and post-traumatic) and failed vasectomy reversal with epididymal obstruction.

**The techniques are as follows:**

- End-to-end anastomosis;
- End-to-side anastomosis;
- Three-suture triangulation intussusception;
- Two-suture transverse intussusception vasoepididymostomy (DIVE);
- Longitudinal intussusception vasoepididymostomy (LIVE).

**Longitudinal Intussusception Vasoepididymostomy (LIVE)**

It includes two techniques: two-suture double-armed longitudinal intussusception vasoepididymostomy (DA-LIVE) and single-armed longitudinal intussusception microsurgical vasoepididymostomy (SA-LIVE).

Advantages of these techniques include a larger anastomotic diameter (reduces stenosis), better fluid flow, improved patency rates, that it is easier to perform than conventional techniques, and better pregnancy rates [241].

**Transurethral Resection of Ejaculatory Duct (TURED)**

It is indicated in patients with ejaculatory duct obstruction (EDO). The procedure involves a transurethral approach using a resectoscope, identifying verumontanum, resecting tissue overlying ejaculatory ducts, opening obstructed ducts into the prostatic urethra and injecting methylene blue into the vas to confirm duct location.

The outcomes are as follows:

- Improvement in semen parameters.
- Spontaneous pregnancy rates reported.

Complications noted in 10–20% and include watery ejaculation, hematuria, epididymitis and infections [242].

**Robot-Assisted Microsurgical Varicocelectomy**

This technique utilizes robotic systems (e.g., da Vinci) providing high-definition 3D visualization and enhanced microsurgical precision.

Advantages include superior visualization (3D and high definition), enhanced dexterity and precision, reduced tremor and potential for better vessel preservation

The outcomes are as follows:

- Improvement in semen parameters comparable or superior to conventional microsurgery.
- Low complication rate.

Limitations include higher cost, the requirement of specialized equipment and training, and limited availability [243].

#### 3.4.11. Assisted Reproductive Technologies (ARTs)

The goal of ARTs is to segregate functional spermatozoa that are competent to fertilize oocytes [244]. Indications by total motile sperm count and clinical context are as follows:

- **Intrauterine Insemination (IUI):** total motile count > 5 million and post-wash sperm count > 1 million; advancing male or female age negatively affects pregnancy outcomes [245].
- **In vitro fertilization (IVF):** total motile sperm count 0.2–1 million, prior IUI failure, or moderate-to-severe male infertility [246].
- **Intracytoplasmic sperm injection (ICSI)**: The advent of ICSI treatment has transformed male infertility treatment and improved reproductive outcomes. Although IVF and other related techniques have been practiced in the past for male infertility, most ART centers now practice ICSI as a primary modality to manage male infertility [247]. The research communities have given various criteria for the selection of suitable patients for ICSI. In general, these criteria include severe oligospermia, <5% progressive motility, <4% normal morphology, use of cryopreserved sperm, the presence of anti-sperm antibodies and failed IVF procedures in the past [248]. For patients with non-obstructive azoospermia, the microdissection testicular sperm extraction (micro-TESE) technique offers higher sperm retrieval success rates and better ICSI outcomes than conventional methods [249].
- Experimental and Emerging ART Techniques
  Round Spermatid Injection (ROSI)
  ROSI is an ART technique in which immature post-meiotic haploid round spermatids are injected directly into the oocytes. It is a rescue option when no mature spermatozoa are found during TESE in non-obstructive azoospermia.
  After ejaculation or TESE extraction, round spermatids are identified (morphological criteria and immunostaining) and injected into oocytes. Artificial oocyte activation is often required.
  The challenges are as follows:
  - Difficult to distinguish round spermatids from diploid spermatocytes.
  - Requires expert embryologist.
  - Phase contrast microscopy and fluorescent mitochondrial probe may be required.
  - May have impaired oocyte activation capacity.
  - Concerns regarding incomplete epigenetic reprogramming and imprinting.
  - Potential genetic and epigenetic risks remain under investigation.
  The outcomes are as follows:
  - Overall fertilization rates (45–50%) are lower than ICSI.
  - ROSI is limited in clinical practice and is not recommended for routine clinical use.
  - May be considered in research settings with full ethical approval, informed consent and long-term offspring follow-up [250].
  **The aspects of elongated spermatid injection (ELSI) are as follows:**
  - Utilizes elongated spermatids, which are more mature than round spermatids.
  - Fertilization and pregnancy outcomes appear superior to ROSI.
  - Identification is easier due to characteristic morphology [251].
  **Clinical Counselling**
  ROSI should not be offered as routine treatment. Patients with non-obstructive azoospermia and failed TESE should receive counselling regarding realistic reproductive options, including donor sperm, adoption, or remaining child-free-, as discussed in Section 3.4.9.

#### 3.4.12. Advanced Sperm Selection Techniques for ART

Emerging technologies aim to optimize sperm selection beyond density gradient centrifugation and swim-up methods.

**Intracytoplasmic Morphologically Selected Sperm Injection (IMSI)**

IMSI utilizes ultra-high magnification (~6000×) with differential interference contrast optics and digital enhancement to evaluate fine sperm morphology prior to ICSI. This allows visualization of nuclear vacuoles, acrosomal integrity, and subtle structural abnormalities not detectable at conventional magnification [252].

Nuclear vacuoles correlate with DNA fragmentation, and more vacuoles are associated with increased DNA damage, lower pregnancy rates and higher miscarriage rates.

The indications are as follows:

- Repeated ICSI failures;
- High sperm DNA fragmentation;
- Recurrent pregnancy loss.

Evidence of outcomes remains controversial. Some studies report improved embryo quality and pregnancy rates, whereas meta-analyses suggest limited benefit over conventional ICSI. Selected subgroups (e.g., high DNA fragmentation, repeated ART failure) may benefit.

Limitations are as follows:

- Time-consuming and requires expensive equipment and expertise.
- Limited availability and increased oocyte handling time.
- Not universally beneficial [253,254].

Current recommendations are as follows:

- Consider selected cases with repeated failures or high DFI;
- Not recommended as routine replacement for standard ICSI;
- More research is needed to define optimal patient selection.

**Microfluidic Sperm Selection**

Microfluidic sperm selection mimics physiological sperm migration within the female reproductive tract, enabling isolation of motile sperm with superior DNA integrity [255].

Advantages over conventional methods are as follows:

- Physiological selection: mimics natural selection.
- Avoids centrifugation-induced oxidative stress.
- Rapid with minimal sperm manipulation [256].

**Microfluidic Sperm Sorting Devices**

**(a) Zymot (ZyMot Multi, ZyMot ICSI)**

This is a multi-chamber microfluidic device where sperm swim through membrane with pores and motile sperm with intact membranes pass through, whereas dead sperm, debris, and leukocytes are excluded.

Reported benefits include

- Improved motility and morphology.
- Lowers DNA fragmentation.
- Improved fertilization rates, implantation rates and pregnancy rates.

**(b) FERTILE Chip:**

This is a microfluidic device with dual chambers separated by a microporous membrane. Motile sperm migrate through microchannels, enabling the selection of sperm with normal morphology and intact DNA [256]. Descriptions of some techniques are as follows:

- **Hyaluronic acid binding:** This technique selects viable non-apoptotic sperm devoid of DNA fragmentation and reduces the miscarriage rate when compared to standard ICSI [257].
- **Magnetic-activated cell sorting (MACS):** This technique removes apoptotic sperm and leads to improved pregnancy outcomes [255].
- **AI-enhanced morphology selection:** This includes multi-sperm tracking algorithms capable of accurately measuring sperm motility [258].
- **Automated DFI-integrated selection:** These combine conventional parameters with DNA integrity, and their use has the potential to improve pregnancy rates [259].

#### 3.4.13. Varicocelectomy

Varicocele is one of the common and surgically correctable causes of male infertility [260,261]. Although there have been plenty of debates regarding the successful achievement of conception among post-varicocelectomy patients, several studies reported that varicocele correction significantly improved the semen parameter values and led to increased pregnancy rates [262,263,264]. However, the greatest benefit is observed in men with palpable varicoceles, abnormal semen parameters, and no significant female infertility factors [265].

Varicocelectomy may also reduce the DNA damage of sperm mediated by hyperthermia and oxygen free radicals [266]. Varicocele interventions are generally categorized by being classified into two main approaches: surgical and non-surgical/radiological. Surgical varicocelectomy is further classified based on the access and approaches for varicocele repairs. These surgical approaches include open varicocelectomy (inguinal, retroperitoneal, or scrotal), laparoscopic varicocelectomy, and microsurgical techniques [267,268]. The radiological approaches are antegrade or retrograde embolization and sclerotherapy. The benefits of radiological procedures include less invasiveness and repair of even small veins that surgical procedures may not detect. The radiological procedures may provide an excellent alternative to surgical procedures, as the complication rate is low and outcomes are identical [269,270].

A recent meta-analysis conducted by Birowo et al. in 2020 revealed that varicocele repair done among infertile males through different surgical procedures is significantly associated with higher pregnancy and live birth rates [271]. Notably, a systematic review by Kim HJ et al. 2016 found that varicocele repair in patients with subclinical varicocele did not result in a significant improvement in pregnancy rates [272]. These reports are essential to consider the patient’s selection for varicocelectomy.

**Secondary Varicocele**

Secondary varicocele results from the obstruction of testicular venous drainage by extrinsic compression, rather than primary valvular incompetence.

The causes include

- Renal cell carcinoma and renal vein thrombosis.
- Retroperitoneal tumors (lymphoma, sarcoma) or retroperitoneal fibrosis.
- Inferior vena cava obstruction.
- Liver cirrhosis caused by portal hypertension [273].

Diagnostic workup includes detailed history and physical examination, abdominal/pelvic ultrasound, Doppler color flow, CT scan, venography and tumor markers if indicated [274].

Management includes the following:

- Treat the underlying cause; varicocele may resolve after primary pathology is addressed.
- Varicocelectomy should be deferred until malignancy or vascular obstruction is excluded.
- Oncologic management takes priority when tumors are identified.
- Fertility preservation (e.g., sperm cryopreservation) should be considered before cancer therapy.

Clinical significance is as follows:

- Secondary varicocele may be presenting sign of serious pathology.
- Imaging is mandatory before varicocelectomy in men >40 years with new varicocele, right-sided or bilateral varicocele and varicocele with systemic symptoms
- Failure to recognize secondary causes may delay diagnosis of life-threatening conditions.

#### 3.4.14. Lifestyle and Its Related Factor Modifications

These multiple factors could negatively impact sperm parameter quality and ROS-induced sperm DNA damage [79,275]. Modifying these multiple lifestyle factors could improve infertile male reproductive health and sperm quality [276].

#### 3.4.15. Mediterranean Diet and Male Reproductive Health

The Mediterranean diet, characterized by high intake of fruits and vegetables, whole grains, nuts and seeds, seafood, and olive oil and low consumption of processed and saturated fats, has been associated with improved reproductive health. It provides essential nutrients and bioactive compounds with strong antioxidant and anti-inflammatory effects that support spermatogenesis and overall male health [277].

**Mechanisms supporting male fertility are as follows:**

- **Antioxidant protection:** The Mediterranean diet is extremely rich in antioxidants like vitamins C and E, polyphenols, carotenoids and flavonoids, which protects sperm from ROS damage and reduces DNA fragmentation.
- **Anti-inflammatory effects:** Omega-3 fatty acids and polyphenols reduce systemic inflammation and improve testicular environment.
- **Hormonal balance:** Healthy fats support testosterone production and zinc and selenium support hormonal function.
- **Vascular health:** This improves cardiovascular function and enhances blood flow to reproductive organs, supporting erectile function and testicular perfusion.
- **Weight management:** The Mediterranean diet promotes healthy weight, reduces obesity-related infertility and improves insulin sensitivity.
- **Epigenetic benefits:** Folate and polyphenols may support normal DNA methylation and gene regulation, promoting healthy sperm epigenetic programming.

**Clinical Perspective**

The Mediterranean diet represents an evidence-based, holistic approach to male reproductive health. Being rich in antioxidants, anti-inflammatory compounds, and essential nutrients, it improves semen quality, protects against environmental damage, and supports overall health. Clinicians should actively counsel infertile men on adopting Mediterranean dietary patterns as a foundational lifestyle intervention [277]. Various integrative initiatives/models are discussed in detail in Section 4.

Dietary patterns represent a modifiable factor that may counteract oxidative stress, metabolic dysfunction, and environmental reproductive toxicity.

#### 3.4.16. Sperm Cryopreservation: Medical and Social Indications

Sperm cryopreservation (sperm banking) is a critical fertility preservation strategy with expanding indications. Semen samples are obtained by masturbation (preferred) or surgical retrieval when necessary. Slow freezing, rapid freezing, and vitrification methods are employed. Post-thaw outcomes include a high motility rate of sperm and high pregnancy rates [278].

**Medical indications are as follows:**

**1. Oncological conditions:**

- Prior to chemotherapy and radiation therapy.
- High-risk cancers for fertility include testicular cancer, Hodgkin and non-Hodgkin lymphoma, leukemias, sarcomas and pelvic malignancies [279].

**2. Non-malignant medical conditions:**

- Autoimmune diseases that require immunosuppressants like SLE and IBD [278].
- Hematological disorders like sickle cell disease (before stem cell transplant), aplastic anemia and thalassemia [280].

**3. Surgical and traumatic risks:**

- Bilateral orchiectomy.
- Pelvic surgery with nerve injury risk
- Gender affirmation surgery (transgender women).
- Testicular torsion or severe trauma [281].

**4. Progressive testicular failure:**

- Klinefelter Syndrome
- Y chromosome microdeletions and XX Male Syndrome.
- Varicocele and cryptorchidism [281].

**5. Assisted reproduction:**

Cryopreservation is an important and a crucial step in assisted reproductive technology (ART). Indications include severe oligozoospermia: ejaculatory dysfunction and azoospermia [278].

**Social (Elective) indications are as follows:**

**1. Delayed parenthood:**

“Social sperm freezing” is analogous to egg freezing for women. Men choosing to delay fatherhood or preserve younger, higher-quality sperm [282].

**2. High-risk professions:**

Military (combat deployment), law enforcement and hazardous occupations with mortality risk [278].

**3. Gender affirmation** [283].

**4. Advanced paternal age concerns (>40-years):**

Preserve sperm before age-related decline and reduce offspring genetic risks associated with aging [284].

**Clinical Considerations**

Sperm cryopreservation is safe and effective, and counseling should be offered to all reproductive-age males at risk of future infertility.

#### 3.4.17. Complementary and Alternative Medicine (CAM) for Male Infertility

Complementary and alternative medicine approaches are increasingly utilized by infertile couples, often alongside conventional treatments. Evidence quality varies, and these therapies should be considered adjunctive rather than primary treatments.

**1.**  **Acupuncture**

Acupuncture is generally safe and may be used as an adjunct therapy.

**Evidence for this treatment is as follows:**

- Some systematic reviews and meta-analyses reported improvements in sperm concentration, motility, and morphology [285,286].
- Warm acupuncture improves sperm concentration, whereas electroacupuncture enhances the sperm motility [286].
- Evidence remains inconsistent, and further randomized trials are needed.

**2.**  **Herbal Medicine**

Herbal therapies are proposed to improve male fertility through antioxidant, anti-inflammatory, and hormone-modulating effects. The beneficial effects are thought to arise from diverse phytochemical constituents such as saponins, phytosterols, carotenoids, phenolic compounds, and alkaloids [287].

**Traditional Chinese Medicine (TCM) formulations are as follows:**

**Wuzi Yanzong Wan:**

- Used in oligoasthenozoospermia.
- Some studies show improved sperm count and motility as well as reduced DNA fragmentation [288].

**Maca:**

- RCTs depicted mixed results, some showing improvements in semen quality parameters, whereas others failed to show the desired effects [289].

**Traditional Persian medicine:**

- Reliable evidence has been obtained for improving sperm abnormalities for some plants, like Chlorophytum borivilianum, Nigella sativa, Sesamum indicum, etc.
- However, further research is needed to determine their safety and efficacy [287].

**Safety concerns:**

- Variability in product quality and dosing.
- Drug interactions: herbs can interact with medications.
- Risk of contamination or toxicity.

**3.**  **Mind–Body Therapies**

Stress reduction therapies such as yoga and meditation may improve psychological well-being and reduce stress-related hormonal effects. Evidence for direct fertility improvement is limited [275].

**4.**  **Other CAM approaches**

They include massage Therapy, homeopathy and Ayurveda.

**Clinical considerations are as follows:**

- CAM should be discussed with healthcare providers to avoid interactions or unsafe practices.
- Patients should use reputable, quality-controlled products.
- CAM should be used as an adjunct to evidence-based medical therapy.

CAM use is common among infertile couples, but high-quality studies evaluating pregnancy and live birth outcomes are still needed.

#### 3.4.18. Emerging and Experimental Therapies

- **Stem cell-based approaches and in vitro spermatogenesis:** Successes have been achieved in animal models and in non-human primates. Although preliminary human research is underway, effective clinical translation will depend on addressing substantial scientific, ethical, and regulatory challenges. Several potential strategies that may be adopted include the following.

**In testicular tissue grafting,** intact testicular tissue fragments are transplanted, thereby preserving the native environment of seminiferous tubules. This may be particularly important for cryptorchid boys with high infertility risk, where cryopreservation of testicular tissue can be done for future grafting [290].

**In testicular tissue organ culture,** intact testicular fragments are maintained in culture to promote germ cell maturation, thereby supporting full spermatogenesis in vitro without relying on grafting. A major advantage is eliminating the potential for introducing malignant cells or xenobiotic contaminants inherent in transplantation techniques [291].

**Testicular cell reconstitution** represents an innovative strategy focused on re-establishing testicular architecture and function using dissociated testicular cells. This approach has been tested across several species, including humans, but the overall efficiency of spermatogenesis remains limited [291].

**Spermatogonial stem cell (SSC) transplantation** involves the transplantation of autologous or donor SSCs into seminiferous tubules and has proven success in animal studies and ongoing exploration in humans [291].

**Pluripotent stem cell-based IVG** involves pluripotent stem cells being used in culture for sperm production; success has been achieved in mouse models [292].

**The testis-on-chip platform** involves ex-vivo testicular tissue cultures in a microfluidic device [293].

- **Gene Therapy and CRISPR-Based Interventions**

CRISPR/Cas9 has largely overcome the limitations of earlier gene-editing tools, which were too complex and inefficient for reliable SSC modification. Success has been achieved in mouse SSC models. Correction of monogenic spermatogenic defects (SPATA16, DPY19L2, *Tsga10*, etc.) may be possible by using gene-editing technologies [294]. Challenges include genetic heterogeneity, delivery to spermatogenic cells, and ethical and regulatory barriers [295].

- **Pharmacological Innovations**

**In long-acting recombinant FSH (Corifollitropin alfa),** a single injection replaces 7 days of daily FSH; it is a potent inducer of multiple follicular growth before IVF and is under investigation for male infertility [296].

**Kisspeptin therapy involves a** GnRH secretagogue; it restores pulsatile GnRH in Hypogonadotropic Hypogonadism, and preliminary studies show promise [297].

## 4. Critical Analysis of Current Trends, Knowledge Gaps, and Future Research Directions for Male Infertility

**The current trends are as follows:**

- **Genetic and molecular understanding:** The latest trends have focused on unraveling the genetic and molecular causes of male infertility. Novel gene mutations have been implicated in impaired spermatogenesis, enhancing both diagnostic and therapeutic potential, with the latest research focusing on multi-omics molecular profiling.
- **Advancements in assisted reproductive techniques:** Significant progress has been made in assisted reproductive techniques over the past decade, particularly in ICSI and sperm selection methods, and these technologies have now become a mainstay for managing male infertility.
- **Environmental and lifestyle factors:** Environmental and lifestyle influences are increasingly recognized as important contributors to endocrine disruption and impaired male fertility, underscoring the need for a clearer understanding of modifiable external determinants.
- **Ecofoodfertility Network:** This is an innovative research initiative that links environmental quality, diet, and reproductive health. This program monitors semen quality and molecular biomarkers in populations exposed to varying environmental pollutants while evaluating the protective role of the Mediterranean diet and lifestyle.
**Key insights from this initiative include the following:**
  - Men living in highly polluted environments tend to have poor semen quality and increased molecular alterations, including oxidative stress and DNA damage.
  - Adherence to antioxidant-rich dietary patterns, such as the Mediterranean diet, may mitigate reproductive harm done by environmentally exposures.
  - Integrated monitoring of environmental exposure, lifestyle factors, and sperm molecular markers provides a powerful model for reproductive surveillance and preventive strategies.
  This approach highlights the need for interdisciplinary research and supports the development of environmental and public health policies aimed at protecting reproductive health [298].

**The knowledge gaps that still exist are as follows:**

- **Gaps in genetic testing:** Despite tremendous work in genetics, a significant knowledge gap remains in understanding the role of epigenetic factors in male infertility as well as the role of undiscovered genes. Moreover, the extent to which environmental factors modulate epigenetic processes remains to be clearly defined.
- **Idiopathic infertility:** Despite recent advances in the diagnostic evaluation of male infertility, idiopathic cases continue to present a significant challenge in defining targeted therapeutic strategies. Approximately one-third of infertile males show no abnormalities on routine semen analysis, highlighting the potential utility of multi-omics approaches, which nevertheless require validation, standardization, and cost optimization before routine application. Despite substantial advances in the assisted reproductive techniques, a proportion of male infertility cases remain refractory to treatment.
- **Semen analysis variability:** Significant intra-individual and inter-laboratory variability undermines the diagnostic accuracy of semen analysis. AI-enhanced automated platforms may offer improved standardization and reproducibility.
- **Functional assay limitations:** Functional assays such as sperm DNA fragmentation and oxidative stress testing lack methodological standardization and consensus cutoff values, alongside a limited availability of validated biomarkers that predict responsiveness to specific interventions such as varicocelectomy or hormonal therapy.
- **Therapeutic controversies:** Most pharmacological treatments, including antioxidants, FSH, and aromatase inhibitors, lack robust evidence from large-scale randomized controlled trials. Varicocelectomy outcomes also remain debated, with some meta-analyses reporting higher pregnancy rates, whereas the patient-specific benefit is difficult to predict, and there is no consensus on optimal selection criteria. Long-term offspring health outcomes following ICSI, particularly with surgically retrieved sperm, require continued surveillance, though current data are largely reassuring.
- **Translational barriers for emerging technologies:** Stem cell therapies are faced with low germ cell differentiation rates, lack of functional validation, and safety and ethical concerns. The clinical application of gene therapy is limited by substantial genetic heterogeneity, delivery barriers to spermatogenic cells, ethical issues related to germline editing, and rigorous regulatory oversight. AI-driven diagnostics face challenges including data variability, limited training diversity, interpretability, regulatory hurdles, and uncertain cost–benefit profiles. Likewise, multi-omics platforms are constrained by high costs, technical complexity, and lack of clinical validation.
- **Addressing environmental and lifestyle concerns:** Sperm quality transcends individual fertility, serving as a vital biomarker of environmental health and population well-being. The global decline in semen quality reflects wider environmental degradation with significant health implications. Protecting male reproductive health requires addressing environmental, occupational, and lifestyle factors at individual and societal levels.
- **Socioeconomic and global health disparities:** Male infertility disproportionately affects low- and middle-income countries due to limited diagnostic infrastructure, restricted access to costly ART services, cultural barriers such as stigma and gender bias that reduce male participation in care, and major prevention gaps including inadequate cryptorchidism management, untreated STIs, and high occupational or environmental exposures.

**Future Research Directions**

Emerging insights into environmental, molecular and lifestyle influences on male fertility emphasize the need for interdisciplinary research and preventive strategies. Future work should prioritize clinically actionable areas that can improve diagnosis, treatment, and prevention.

- **Precision diagnostics and molecular insights (high priority)**
  Future research should move beyond conventional testing to include advanced genetic panels, epigenetic biomarkers, and functional genomics aiming to uncover causes of idiopathic infertility and support precision medicine approaches.

- **Environmental and occupational risk assessment (high priority)**
  Longitudinal cohort studies incorporating exposure biomarkers and environmental monitoring are needed to establish causal links between pollutants and reproductive dysfunction using longitudinal cohort studies. Population-based semen quality registries integrated with environmental surveillance may strengthen risk assessment.

- **Standardization of advanced diagnostic tools**
  Efforts should focus on standardizing sperm function tests, defining consensus thresholds, and developing algorithms that combine clinical, laboratory, and molecular data to improve diagnostic accuracy and guide treatment selection.

- **Optimization of ART and offspring safety**
  Future studies should refine sperm selection methods, validate microfluidic and AI-assisted technologies, and evaluate long-term health outcomes in children conceived through ART.

- **Lifestyle and preventive interventions**
  Large-scale trials are needed to assess the effects of diet, physical activity, stress management, and complementary therapies as adjuncts to fertility care.

- **Emerging therapies**
  Stem cell therapy, gene-based treatments, and microbiome modulation hold promise for conditions currently considered untreatable but require rigorous clinical validation.

- **Addressing global disparities**
  Research priorities should focus on developing affordable diagnostic tools, expanding access to fertility care, providing culturally sensitive education, and strengthening prevention strategies targeting occupational and environmental risks.

- **Multidisciplinary collaboration**
  Multidisciplinary collaboration and international consortia are vital to standardize research methods, facilitate data sharing, and translate scientific advances into equitable clinical care worldwide.

## 5. Limitations of This Narrative Review

This review has several limitations that should be considered when interpreting its findings:

- First, as a narrative review, it does not follow a systematic review methodology, and no formal quality appraisal of included studies was performed. Therefore, selection bias cannot be entirely excluded, and the conclusions should be viewed as a broad synthesis rather than a quantitative evaluation.
- Second, the evidence across topics is heterogeneous and rapidly evolving. While some areas are supported by randomized trials and meta-analyses, others depend on observational studies or expert opinion.
- Third, much of the available literature comes from high-income countries, which may restrict applicability to resource-limited settings where environmental exposures, healthcare access, and genetic backgrounds differ.
- Fourth, emerging approaches such as metabolomics, microbiome analysis, molecular diagnostics, and advanced sperm function testing remain largely investigational, and their routine clinical applicability requires further validation.
- Fifth, male infertility is a multifactorial condition, and isolating environmental and lifestyle influences is challenging due to confounding variables and limited longitudinal data. Consequently, a substantial proportion of cases remain idiopathic.
- Finally, advanced diagnostic techniques and assisted reproductive technologies may be costly and not universally accessible. Additionally, long-term offspring outcomes and potential transgenerational effects require further investigation.

### Strengths Despite Limitations

Despite these constraints, this review offers a comprehensive synthesis of current knowledge on male infertility. It integrates epidemiological, clinical, molecular, and environmental perspectives, highlights emerging diagnostic and therapeutic advances, and identifies key knowledge gaps to guide future research and clinical practice.

## 6. Conclusions

Male infertility represents a complex, multifaceted reproductive health challenge affecting 20–30% of infertile couples worldwide and carries significant medical, psychological, and socioeconomic ramifications. The field has advanced far beyond traditional reliance on semen analysis, shifting toward comprehensive, multi-layered diagnostics that integrates clinical profiling, advanced imaging modalities, hormonal profiling, genetic and epigenetic testing, and multi-omics molecular characterization. Therapeutic strategies have progressed from largely empirical approaches to precision medicine interventions guided by molecular biomarkers, hormonal classification frameworks, and artificial intelligence-augmented decision algorithms.

Recent advances have substantially enhanced our understanding of key pathophysiological mechanisms, including sperm DNA fragmentation, epigenetic alterations, mitochondrial dysfunction, disruptions in seminal microbiome, CatSper channel anomalies, and influence of environmental endocrine disruptors. Emerging diagnostic biomarkers such as TEX101, AMH, inhibin B, automated DNA fragmentation metrics, metabolomic profiles, and AI-assisted imaging modalities demonstrate promising predictive utility for treatment selection and ART outcomes, though rigorous multicenter prospective validation is required. Therapeutic innovations encompass hormonal stratification frameworks, aromatase inhibitors, advanced sperm selection technologies, and experimental regenerative strategies utilizing stem cells and in vitro spermatogenesis platforms. Pioneering areas like epigenetic research and stem cell therapies appear promising for expanding the treatment options and improving prognosis.

Despite significant advances, substantial knowledge gaps and therapeutic limitations remain. Idiopathic male infertility accounts for approximately 30–40% of cases, highlighting the incomplete understanding of the molecular mechanisms governing spermatogenesis and fertilization. Conventional interventions including varicocelectomy, empirical hormonal therapies, and antioxidant supplementation yield only modest and variable benefits, and robust randomized controlled trials with live birth outcomes as the primary endpoint are scarce. Assisted reproductive technologies, particularly ICSI, have revolutionized outcomes for severe male factor infertility, yet significant proportions of couples, especially those with genetic defects affecting spermatogenesis or severe idiopathic oligozoospermia, continue to lack effective therapeutic or reproductive solutions.

Future strategies for managing male infertility will be shaped by integrated advances in precision medicine, regenerative science, and AI technologies, alongside equitable global health frameworks. Multidisciplinary collaboration among andrologists, endocrinologists, geneticists, bioethicists, and public health experts is imperative to translate scientific advances into tangible clinical benefits. These integrated approaches are vital in optimizing reproductive outcomes and improving quality of life for millions of couples worldwide affected by male factor infertility.

## Figures and Tables

**Table 1:** WHO 2021 guidelines (6th edition) [123] for semen analysis lower fifth percentile (with 95% confidence interval).

Semen Parameters	WHO 2021 (Lowest Fifth Percentile with 95% Confidence Interval)
Semen volume	1.4 mL (1.3–1.5 mL)
Total sperm count	39 million per ejaculation (35–40 million/per ejaculation)
Sperm concentration	16 million/mL
Normal sperm morphology (normal forms)	4% (3.9–4%)
Vitality (live spermatozoa)	54% (50–56%)
Total motility (progressive and non-progressive)	42% (40–43%)
Progressive motility	30% (29–31%)

**Table 2:** Johnsen’s Scoring System.

Score	Histological Criteria
10	Complete spermatogenesis with many spermatozoa, organized epithelium
9	Many spermatozoa present, but disorganized epithelium with marked sloughing
8	Only few spermatozoa present (<5–10 per tubule)
7	No spermatozoa, but many spermatids present
6	No spermatozoa, few spermatids (<5–10 per tubule)
5	No spermatozoa or spermatids; many spermatocytes present
4	No spermatozoa or spermatids; few spermatocytes (<5 per tubule)
3	Only spermatogonia present (no meiotic cells)
2	No germ cells, only Sertoli cells present (Sertoli cell-only syndrome)
1	No cells in tubular lumen; seminiferous tubules show sclerosis

## Data Availability

No new data were generated or analyzed in this study. All information presented is derived from previously published literature.

## Abbreviations

The following abbreviations are used in this manuscript:

ART	Assisted reproductive technologies
WHO	World Health Organization
SDF	Sperm DNA fragmentation
ROS	Reactive oxygen species
FSH	Follicle-stimulating hormone
LH	Luteinizing hormone
GnRH	Gonadotropin-releasing hormone
TSH	Thyroid stimulating hormone
BMI	Body mass index
DFI	Sperm DNA fragmentation index
piRNAs	Piwi-interacting RNAs
hCG	Human Chorionic Gonadotrophins
PESA	Percutaneous epididymal sperm aspiration
MESA	Microsurgical epididymal sperm aspiration
TESA	Testicular sperm aspiration
IUI	Intrauterine Insemination
